# Unraveling the role of MXene (Ti_3_C_2_T_x_) integrated Cu-doped WO_3_ nanocomposites via co-precipitation technique for enhanced supercapacitor performance

**DOI:** 10.1038/s41598-025-10174-z

**Published:** 2025-07-11

**Authors:** T. Jaqulin Jenila, W. Trinisha Infancy, R. Rathikha, P. Annie Vinosha, Manikandan Ayyar, S. Ramasamy, S. Maruthasalamoorthy, R. Navamathavan, Belina Xavier, Abdullah M. S. Alhuthali, Hala M. Abo-Dief, Magda H. Abdellattif, R. Balachandran, M. Khalid Hossain

**Affiliations:** 1 Department of Physics, Presidency College (Autonomous), Affiliated to the University of Madras, Chennai, Tamil Nadu 600005 India; 2 Department of Physics, Stella Maris College, Affiliated to the University of Madras, Chennai, Tamil Nadu 600086 India; 3 Department of Chemistry, Karpagam Academy of Higher Education, Coimbatore, Tamil Nadu 641021 India; 4 Center of Excellence in Advanced Materials and Green Technologies, Amrita School of Engineering, Amrita Vishwa Vidyapeetham (Coimbatore campus), Coimbatore, Tamil Nadu 641112 India; 5 Department of Physics, School of Advanced Sciences, Vellore Institute of Technology (VIT) Chennai, Chennai, 600127 India; 6 Department of Physics, College of Sciences, Taif University, P.O. Box 11099, Taif, 21944 Saudi Arabia; 7 Department of Science and Technology, University College-Ranyah, Taif University, P.O. Box 11099, Taif, 21944 Saudi Arabia; 8 Department of Chemistry, College of Science, University College of Taraba, Taif University, P.O. Box 11099, Taif, Saudi Arabia; 9 Department of ECE, College of Electrical Engineering and Computing, Adama Science and Technology University, P.O.Box No. 1888, Adama, Ethiopia; 10 Institute of Electronics, Atomic Energy Research Establishment, Bangladesh Atomic Energy Commission, Dhaka, 1349 Bangladesh; 11 Department of Advanced Energy Engineering Science, Interdisciplinary Graduate School of Engineering Sciences, Kyushu University, Fukuoka, 816-8580 Japan; 12 Centre for Material Chemistry, Karpagam Academy of Higher Education, Coimbatore, Tamil Nadu 641 021 India

**Keywords:** Copper-doped tungsten oxide (Cu-WO_3_), Ti_3_C_2_T_x_ MXene, Co-precipitation, Electrochemical performance, Specific capacitance, Energy storage, Energy science and technology, Materials science, Nanoscience and technology

## Abstract

The rising population and increased energy consumption drive contemporary researchers to develop highly efficient electrode materials for high-power energy storage devices. Herein, copper-doped tungsten oxide (Cu-WO_3_) and compositing MXene (Cu-WO_3_/MXene) in different concentrations have garnered substantial interest for their usage as an electrode material owing to their impressive energy-storing capacity, including high metallic conductivity, hydrophilic nature, and exceptional electrochemical performance due to their active surface chemistry. In the present work, we employ a facile co-precipitation technique to fabricate WO_3_ and Cu-WO_3_ (Cu x% = 5 at%, 10 at%, and 15 at%). Furthermore, we synthesized a synergistic 15 at% Cu-WO_3_/MXene nanocomposite by integrating Cu-WO_3_ and MXene via sonication. The synthesized sample’s structure, functional, morphology, chemical composition, and electrochemical properties were examined through various techniques such as X-ray powder diffraction (XRD), Fourier transform infrared spectrum (FT-IR), X-ray photoelectron spectra (XPS), Field Emission Scanning Electron Microscopy (FESEM), and High-Resolution Transmission Electron Microscopy (HRTEM). The X-ray diffraction analyses corroborated the monoclinic state of WO_3_ along with the substitutional inclusion of Cu in the WO_3_ lattice integrated with MXene. Utilizing a Field Emission Scanning Electron Microscope (FESEM), the surface morphological analysis revealed the formation of Cu-WO_3_ nanospheres embedded in MXene sheets. Furthermore, according to results obtained from electrochemical analysis profiles, at 1 mA, 15 at% Cu-WO_3_/MXene displayed a greater specific capacitance of 692.4 F/g in comparison to other electrode materials via a three-electrode system, which is due to the synergistic impact of the Cu-WO_3_ as well as the conductive properties of MXene sheets. Also, the electrode demonstrated excellent cycling stability, retaining 89% of its initial capacitance over 5000 charge-discharge cycles. The Ragone plot revealed an energy density of 70.10 Wh/kg at a power density of 809.8 W/kg. B-value analysis and scan rate-dependent CV confirmed the contribution of both surface-controlled and diffusion-controlled charge storage mechanisms. Likewise, in contrast to all other synthesized materials, 15 at% Cu-WO_3_/MXene revealed a lesser solution resistance and charge transfer resistance. In accordance with the results, the 15 at% Cu-WO_3_/MXene nanocomposite is an extremely efficient capacitive material that can enhance electrochemical performance in energy storage applications.

## Introduction

In the past few decades, the world’s ongoing reliance on fossil fuels as a primary energy source has been reflected in both significant ecological issues along the depletion of renewable energy resources. Population expansion, societal advancements, and contemporary lifestyles have raised high requirements for energy resources^1,2^. Beyond 2050, worldwide energy demands are expected to rise by over 10^10^ tons of fossil fuels every single year. Due to the fact that they’re readily accessible and have a high energy density, conventional fuels are a vital energy source. However, the amount of those fuels that are readily available is severely limited, making it difficult for us to adequately satisfy our energy needs going forward. Therefore, the ongoing rise in energy consumption and pollution rates necessitates the development of more affordable, efficient, sustainable, and ecologically friendly energy sources. For this purpose, experts have created energy conversion and storage systems that are both incredibly efficient and affordable^3^.

To ensure that the energy is utilized in a sustainable and ecologically friendly way, electrochemical energy production is being meticulously considered as an alternative energy source. Amongst numerous energy-storing systems, electrochemical energy-based products, including fuel cells, battery cells, and supercapacitors, have been touted to be the most viable for the energy deficit. Longer cyclic stability, effective charge/discharge rates, and extremely high energy and power densities are all characteristics of the perfect electrochemical energy storage system^4–6^. Although fuel cells and batteries offer a high energy density, their exceptionally rapid discharge rate causes their efficiency to drastically decline over time. Conversely, conventional capacitors are susceptible to a low energy density despite having a high power density. In order to overcome the foregoing constraints of the electrochemical devices, supercapacitors are an ideal selection since they span the division between the batteries as well as conventional capacitors^7–12^.

Supercapacitors have piqued the attention of contemporary electrochemical researchers due to their excellent reversibility, cyclic stability, and ecologically friendly characteristics. In accordance with their charge-storing techniques, supercapacitors tend to be classified into three main categories: (i) electrostatic double-layer capacitors (EDLCs), (ii) redox or pseudo-capacitors, and (iii) hybrid capacitors. In EDLC, materials like graphene, activated carbon, carbon aerogels, etc., have been utilized to electrostatically deposit ions at the electrode-electrolyte interface, retaining the charge^13^.

Furthermore, carbon materials have outstanding structural and electrochemical features, including a large surface area, high electrical conductivity, and stability. Pseudo-capacitors store charge through reversible faradaic reactions occurring at the electrode interface, utilizing transition metal compounds, electrically conductive polymers, and carbon-based materials. Yet another form of supercapacitor classification includes hybrid capacitors, which are composed of EDLC materials, various pseudocapacitive materials, and battery-based materials^14^.

Among all the supercapacitors, the pseudo-capacitor stands out as a viable contender to fulfill rising demands by bridging the gap between batteries and supercapacitors by concurrently increasing energy and power densities. Yet, one of those major drawbacks to the extensive application of supercapacitors for electrical energy storage processes is increasing their energy density when in comparison with batteries. Thus, the quest for novel approaches for elevating energy density values in supercapacitors, particularly through the use of advanced electrode materials, has intensified^15^. This approach seems to be one way to achieve high specific capacitance and ultimately high energy density values. In regard to this, choosing the electrode material that is appropriate for various supercapacitor elements is another facet of the pursuit. An appropriate electrode material optimizes the entire synergy of effective interactions amongst the supercapacitor’s constituent components, resulting in enhanced device performance. An optimum electrode material should possess a large surface area, controlled pore structure, appropriate active electrochemical sites, and extremely efficient electronic conductivity^16^.

Herein, transition metal (TM) oxides and transition metal dichalcogenides (TMDs) are among the most promising electrode materials in this case, owing to their multiple oxidation states, high theoretical capacitance, and cost-effectiveness. The utilization of transition metal oxides as electrode materials has resulted in exceptional charge storage capabilities, and alongside these compounds, they provide further advantages that include extensive surface area, high electrical conductivity, a significant presence of structural defects, low toxicity, affordability, and abundance in the environment. Various transition metal oxides have been previously employed for electrode constituents in supercapacitor applications, with excellent outcomes that have been published. In the research literature, oxides such as WO_3_, RuO_2_, MnO_2_, NiO, ZnO, TiO_2_, and CO_3_O_4_ have previously been demonstrated with promising outcomes as they exhibit excellent faradaic charge storage mechanisms, while TMDs such as MoS_2_ and WS_2_ offer high surface area, tunable electronic properties, and mechanical flexibility, making them suitable candidates for hybrid or composite electrode systems. Recent studies have demonstrated significant advancements in TMO and TMD-based supercapacitor electrodes, showcasing improved electrochemical performance through doping, heterostructure formation, and hybridization with conductive materials. For instance, the incorporation of conductive substrates such as MXene or graphene has been shown to enhance charge transport and cycle stability^17,18^.

Tungsten trioxide (WO_3_) has emerged as one of the TM oxides that has garnered a lot of interest for its usage as an electrode material in SCs. In addition to offering benefits including excellent temperature stability, resisting corrosion, numerous valencies, an extensive negative potential range, and natural abundance, WO_3_’s tunnel-like structure assists in the electrolyte’s ion insertion. The above n-type semiconductor has a large pseudo-capacitance, excessive energy, and packing density, and variable oxidation states. Due to its exceptionally high electrical conductivity, which provides an effective charge transfer between the electrode and the electrolyte, WO_3_ has become arguably the most viable electrode material amongst other metal oxides^18^. The material is chemically stable and maintains the crystal structure during multiple charge-discharge cycles, resulting in excellent cycle stability as well as consistent performance over time. WO_3_ exhibits a variety of phases, including orthorhombic, monoclinic, triclinic, tetragonal, etc., and its crystalline structure is primarily influenced by its preparation techniques and temperature. It does, however, exist in its most stable monoclinic phase at ambient temperature. Numerous chemical techniques, such as the hydrothermal process, sol-gel, and chemical deposition approach, have been utilized to fabricate WO_3_ nanoparticles having different morphologies^19^.

The co-precipitation approach stands out amongst the aforementioned preparation techniques due to its homogeneity, low expenditure, high efficiency, moderate temperature, high product purity, and ease of manipulation. At present, binary metal oxides tend to be the most appropriate materials for fulfilling the specifications of pseudo capacitors, which are more active sites, modified surface area, enhanced electrical conductivity, and multiple redox mechanisms. The most basic approach to producing bimetallic materials is doping.

The electrochemical behavior of WO_3_-based supercapacitors is generally considered favorable in research; however, the reported energy density values do not seem persuasive. To improve this aspect and further enhance supercapacitor performance, doping WO_3_ with transition metal ions has been explored as a highly effective strategy^20^. Therefore, doping different elements (for instance, Cu, Fe, Co, or Ni, respectively) into the crystal lattice of the WO_3_ matrix will have an impact on its crystal structure, subsequently altering its morphology and electrical structure and resulting in a more stable electronic state within the energy band along with a narrower band gap. Herein, the copper (Cu) TM ion that exists in the WO_3_ lattice affects the oxygen vacancies, which might result in phase transformation and change the charge transport properties. Several investigations on Cu-doped WO_3_ as an electrode material for SCs showed promising outcomes. Along with this, MXene, a 2D material, has been employed in conjunction with Cu-doped WO_3_ to even further enhance the synthesized nanoparticles, thereby achieving remarkable performances for supercapacitor applications^21^.

A novel class of two-dimensional elements known as MXenes has recently received focus owing to its potential uses in rechargeable batteries, supercapacitors, electromagnetic wave shielding, and several other applications. It is regarded as one of the most significant feasible electrode compounds for ultra-high supercapacitors owing to its metallic electrical conductivity, high mechanical strength, huge surface area, structural stability, and hydrophilic properties^22^. MXenes are a type of 2D transition-metal carbide, nitride, and carbonitride that are predominantly synthesized from Mn + 1AXn (MAX) predecessor materials, wherein M symbolizes the transition metal, A tends to be a III or IV group element, X represents carbon and even nitrogen, where *n* = 1, 2, or 3. They are prepared by etching “A” layers from the stacked carbides or carbonitrides in the MAX (Ti_3_AlC_2_) phase. To obtain MXene, selectively etching it will be an essential step. Acid-salt variations, which include pure hydrofluoric acids (HF), HCl + LiF (producing HF in situ), and HF + HCl, among others, are utilized to effectively etch the materials. Therefore, introducing pseudocapacitive elements, such as metal oxides, onto MXenes will enhance their electrochemical energy storage properties. Furthermore, this study looked into MXene intercalated with Cu-doped WO_3_ nanoparticles, which functioned as a positively charged electrode, and activated carbon (AC) was used as a negatively charged electrode to create a supercapacitor device^23^.

Thereby, among transition metal oxides, tungsten trioxide (WO_3_) has garnered attention as a pseudocapacitive electrode material due to its multiple valence states (W^6+^/W^5+^), low toxicity, and relatively high theoretical capacitance. However, pristine WO_3_ often suffers from poor electronic conductivity and limited ion diffusion. Doping WO_3_ with transition metal cations (e.g., Cu^2+^) has been shown to introduce oxygen vacancies, narrow the bandgap, and enhance electrical conductivity, leading to improved specific capacitance and rate performance. Meanwhile, 2D transition-metal carbides/nitrides (MXenes), such as Ti_3_C_2_T_x_, exhibit metallic conductivity, high surface area, and hydrophilic surface terminations, which make them excellent conductive scaffolds for pseudocapacitive oxides. By anchoring Cu‐doped WO_3_ nanorods onto an MXene matrix, one can combine the high pseudocapacitance of doped WO_3_ with the fast electron transport and mechanical flexibility of MXene, addressing both conductivity and stability drawbacks simultaneously^24^.

According to this present research, we demonstrate the co-precipitation approach for synthesizing WO_3_ and Cu-doped WO_3_ (Cu x% = 5 at%, 10 at%, and 15 at%), as well as their composite with MXene (Cu-WO_3_/MXene) via a facile sonication process. To significantly enhance the material’s electrical conductivity along with providing a broad surface area, CuWO_3_/MXene was fabricated. Herein, we report high-performance, next-generation supercapacitor devices assembled using a combination of MXene. Considering the observed data, the CuWO_3_/MXene nanocomposite exhibits a high specific capacitance, rendering it an extremely efficient material for implementation in supercapacitor applications^25^.

## Experimental section

### Materials and methods

Every single reagent that was utilized in the reaction was analytically graded and had been used without any further refinement. Sodium tungstate dihydrate (Na_2_WO_4_•2H_2_O) (base precursor) was procured from Sigma Aldrich Pvt. Ltd. with 99.99% purity; copper (II) nitrate dihydrate (Cu(NO_3_)_2_•2H_2_O) (99% reagent grade); concentrated hydrochloric acid (HCl); MAX powder (Ti_3_AlC_2_) (98% purity); lithium fluoride (LiF, ≥ 98%), along with deionized water (DI), were utilized to carry out the analysis. All of the necessary chemicals were procured from Merck Life Science Pvt. Ltd. with 99% purity and are used for the synthesis of CuWO_3_/MXene Composites.

### Synthesis of pure and Cu-doped WO_3_ nanoparticles

**Fig. 1:**
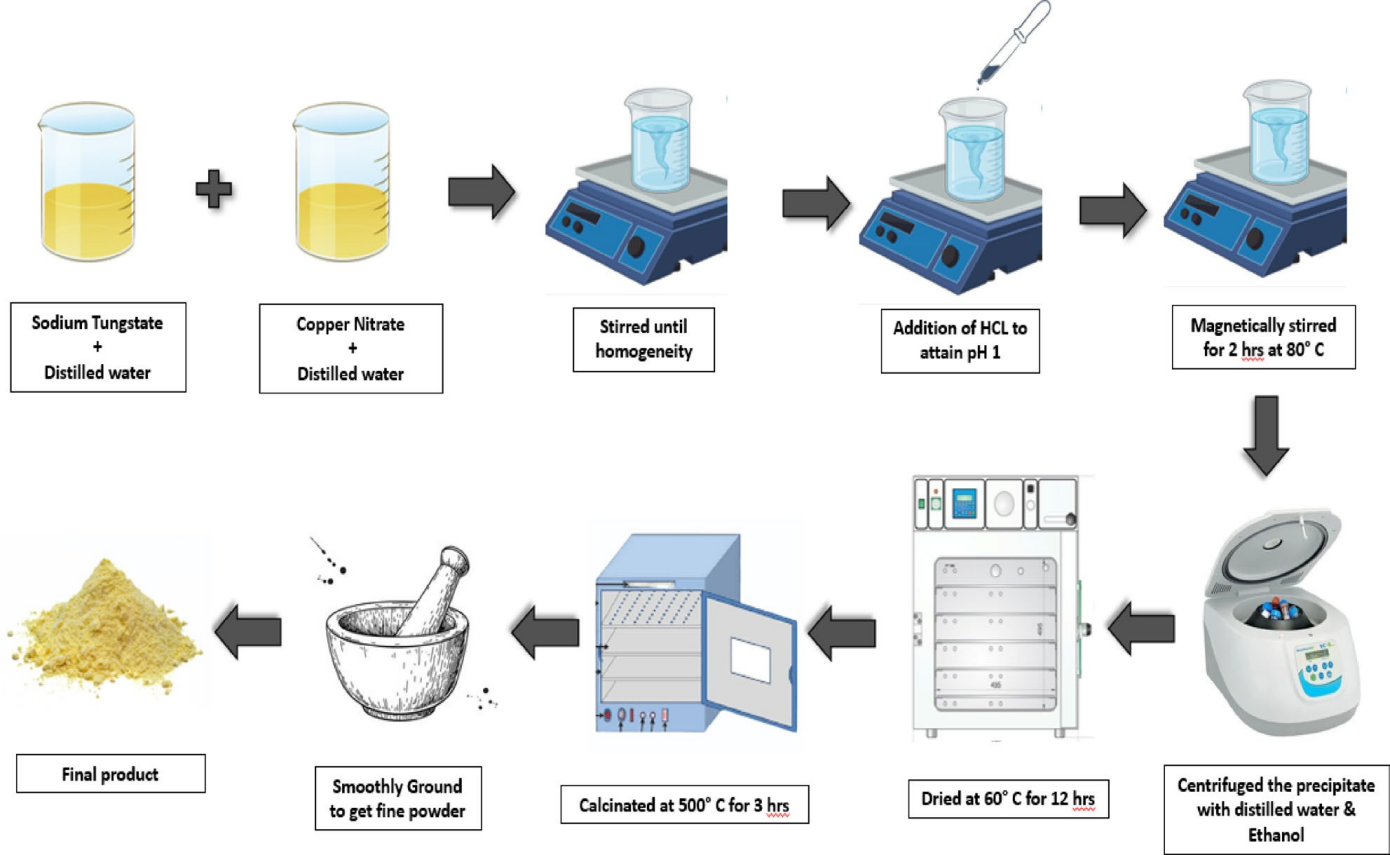
Schematic representation summarizing the synthesis of Cu-WO_3_ nanoparticles.

The tungsten oxide (WO_3_) nanoparticles were synthesized utilizing a co-precipitation technique (Fig. 1). To prepare the solution, 0.5 M sodium tungstate dihydrate (Na_2_WO_4_•2H_2_O) was completely dispersed in 40 mL of DI water (deionized). The resulting solution is vigorously agitated at ambient temperature for 30 min in order to attain homogeneity prior to being acidified with hydrochloric (HCl) acid. Under continuous stirring, about 5 mL of concentrated HCl was added dropwise into the clear solution until the pH was maintained constant at pH 2. After a few hours of stirring, the solution eventually turns into pale yellow precipitates, which indicates the presence of tungstic acid. The aqueous mixture was then subjected to heat for 2 h at 80 °C, cooled to room temperature, and left undisturbed overnight to cool and settle. The aqueous phase was extracted, and the residual precipitate was centrifuged at 5000 rpm with ethanol and distilled water several times to remove excessive sodium and chlorine ions, after which the byproduct was dried for 12 h at 60 °C. Finally, the ensuing powder was annealed at 500 °C for 3 h, and the end product was ground using an agate mortar and pestle. Furthermore, Cu-doped WO_3_ nanoparticles were synthesized via the same co-precipitation method, by adding the required molar concentration (x% = 5 at%, 10 at%, and 15 at%) of copper (II) nitrate dihydrate (Cu(NO_3_)_2_•2H_2_O) in the initial aqueous solution (illustrated in Fig. 1). The above process was carried out, resulting in a pale green Cu-WO_3_ nano powder. The reaction is represented as follows^26^:

$${\text{Na}}_{2} {\text{WO}}_{4} \cdot 2{\text{H}}_{2} {\text{O}} + 2{\text{HCl}} \to {\text{WO}}3 + 2{\text{NaCl}} + 3{\text{H}}_{2} {\text{O}}$$

### Synthesis of MXene

MXenes (Ti_3_C_2_T_x_) are typically formed by selectively etching out the A (aluminum) layers from the precursor MAX (Ti_3_AlC_2_) phases. Initially, at room temperature, 4 M LiF was gradually dispersed in 20 ml of 9 M HCl solution in a Teflon vessel, agitating for about 15 min. In order to prevent a strong exothermic reaction, 0.5 g of Ti_3_AlC_2_ (MAX) powder was slowly added to the solution, pinch by pinch. Subsequently, for maximal etching of the Al layer, the Teflon-containing solution was held in a water bath that had been maintained at 60 °C and magnetically agitated for around 48 h inside a fumigation chamber. Following the reaction, the multi-layer MXene (Ti3C2) was obtained by centrifugation at 6000 rpm with DI water and ethanol repeatedly until the pH level of the solution reached around 6. The resulting byproduct was subsequently air-dried for 25 h at 80 °C within a vacuum oven^27,28^. The general chemical process is represented as follows:

$${\text{Ti}}_{3} {\text{AIC}}_{2} + {\text{HCl}} + 3{\text{LiF}} \to {\text{Ti}}3{\text{C}}_{2} + {\text{AlCl}}_{3} + 3{\text{LiCl}} + 3/2{\text{H}}_{2}.$$

### Synthesis of CuWO_3_/MXene composites

**Fig. 2:**
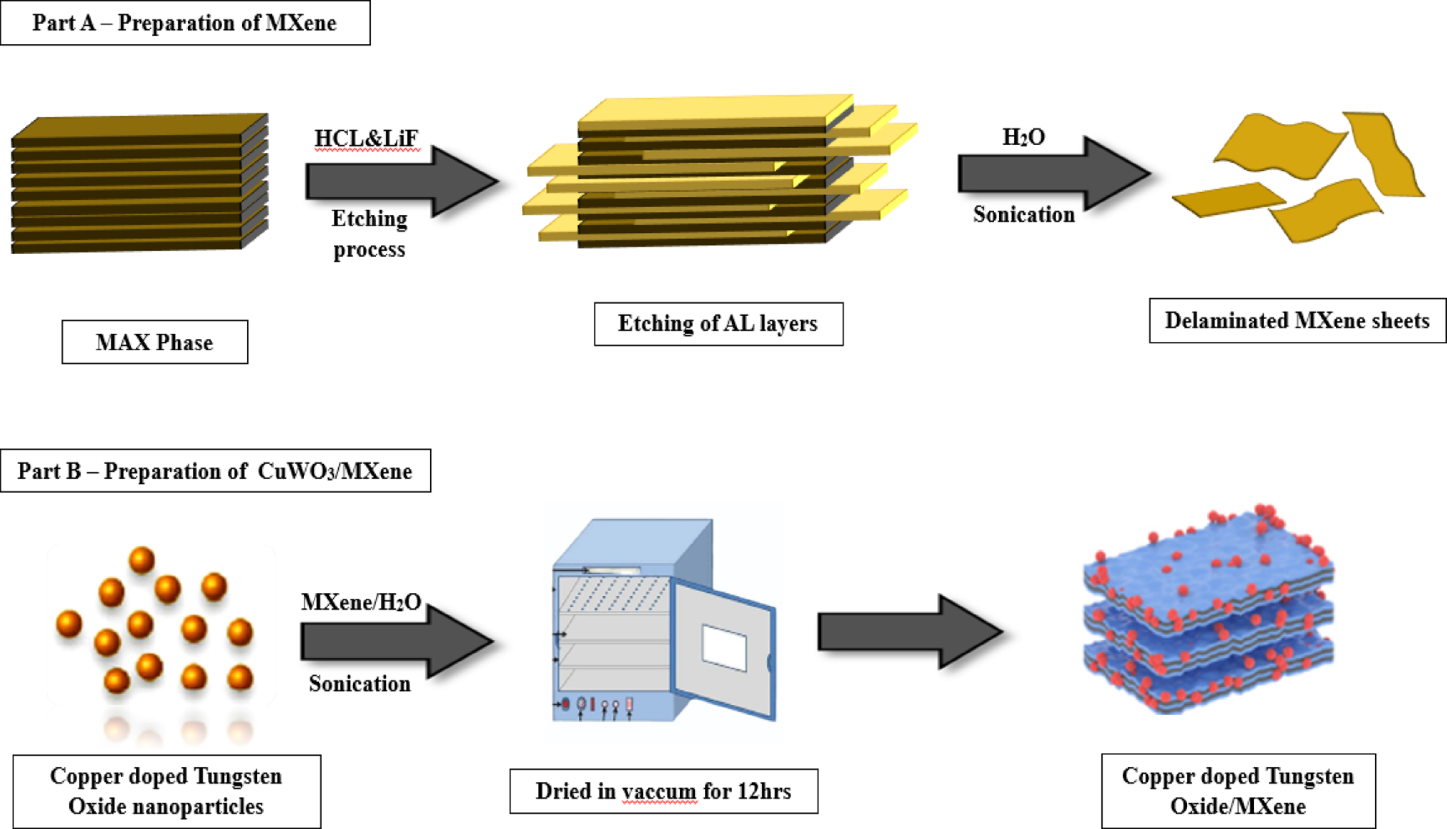
Schematic illustration summarizing the synthesis of MXene and CuWO_3_/MXene nanocomposite.

A simple ultrasonication process was employed to fabricate CuWO_3_/MXene Composites (1:1), which is depicted in Fig. 2. About 0.5 g of the as-synthesized CuWO_3_ NPs and 0.5 g of MXene were dissolved in 30 mL of DI water and sonicated for a period of 2 h, after which the precipitate was homogenized, resulting in a well-disseminated solution. The ensuing product underwent a drying process in a hot air oven at around 70 °C for 12 h. In the end, the dried product was pulverized with an agate mortar and pestle and utilized to perform electrochemical measurements^29^.

### Electrochemical analysis

The electrochemical investigations, which include galvanostatic charge-discharge (GCD), cyclic voltammetry (CV), and electrochemical impedance spectroscopy (EIS), of the as-synthesized CuWO_3_/MXene nanocomposites were carried out on a Biologic SP-300 electrochemical analyzer workstation with the standard three-electrode system. In this study, a saturated calomel electrode (SCE) and a platinum wire were deployed as the reference and counter electrodes in a 1 M KOH electrolytic solution at ambient temperature. Employing the doctor blade method, the working electrode was set up with the as-synthesized material serving as the active material and carbon black functioning as a conductive mediator amalgamated along with the PVDF binder in an 80:10:10 ratio. Following that, N-methyl pyrrolidone (NMP) was added dropwise to homogenize the mixture. The resultant slurry was subsequently coated on 1 × 1 cm^2^ of Ni-foam and dried out for 12 h at 60 °C in an oven^30^.

### Material characterization

In order to analyze the crystalline phase and structure of the synthesized material, X-ray diffraction (XRD)^31–34^ was carried out using Bruker AXS D8 advanced equipment with Cu Kα radiation (λ = 0.15418 nm). The XRD spectrum, comprised of 2θ values, was measured from 5°to 80° at a scanning rate of 5°/s. The Perkin Elmer FTIR spectrophotometer was employed to examine the functional group’s chemical structure and bonding on the material’s surface. The morphological characteristics of the surface of the as-synthesized material have been analyzed using field emission scanning electron microscopy (FE-SEM), which was carried out on a JEOL JSM IT-800 scanning electron microscope^35^. In addition, Energy-Dispersive X-ray spectroscopy (EDX) in a scanning electron microscope was performed to examine the element composition. Furthermore, the JEOL JEM-3010 plus electron magnifier equipment operated at 100 kV has been employed to capture high-resolution (HR-TEM) images. The material’s oxidation states and binding energy were ascertained utilizing X-ray photoelectron spectroscopy (XPS) (Model: Versa Probe III). A Gaussian distribution technique was implemented to deconvolute and fit the XPS data. The electrochemical measurements were carried out utilizing a three-electrode setup on the Biologic SP-300 electrochemical workstation^36^.

## Results and discussion

### X-ray diffraction (XRD)

**Fig. 3:**
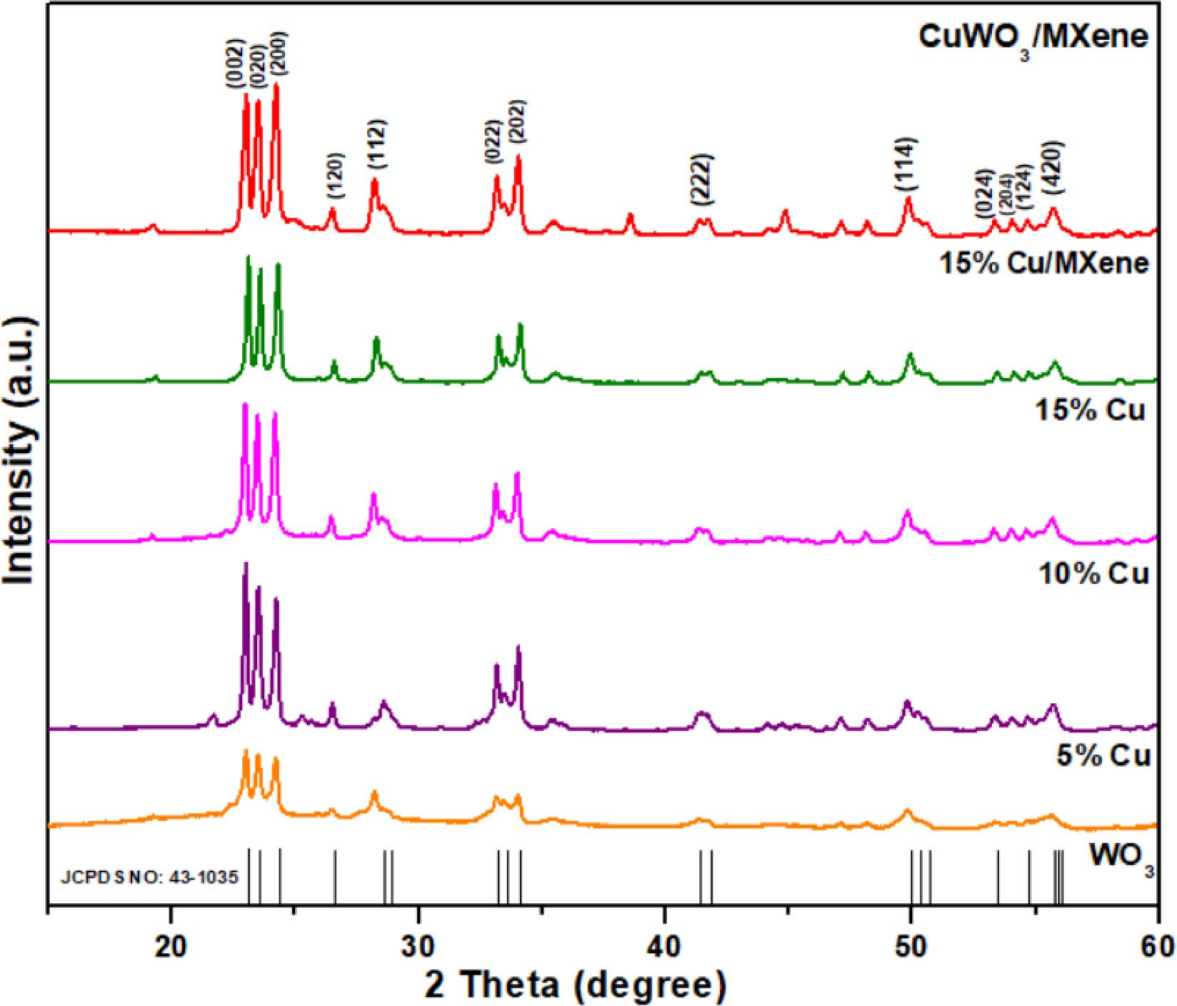
XRD spectra of Pure WO_3_, Cu-WO_3_ (Cu x% =5 at%, 10 at%, 15 at%) and 15 at% Cu-WO_3_/MXene nanocomposite.

By utilizing an XRD equipped with Cu Kα radiation as the source (λ ≈ 1.5406 Å), the material’s crystalline structure, phase purity, crystal size, and space group, that of the as-synthesized WO_3_, Cu-doped WO_3_, MXene, and their composite (Cu-WO_3_/MXene), were investigated. The X-ray diffractograms are illustrated in Fig. 3. The XRD analysis is configured with the following specifications: voltage = 30 kV, current = 20 mA, scanning speed = 2 °/min, and scanning range: 5° to 80°^37^. Figure 3 depicts the XRD spectrum of: Cu-doped WO_3_ (Cu x% = 0 at%, 5 at%, 10 at%, 15 at%) nanoparticles that have been annealed at 500 °C. The two theta degrees at 23.0°, 23.5°, 24.2°, 26.4°, 28.2°, 33.2°, 34.0°, 41.5°, 47.1°, 48.1°, 49.8°, 53.3°, 54.0°, 54.7° and 55.7° correlate with the corresponding diffracted peaks of the tungsten oxide (WO_3_). The aforementioned peak configurations have Miller indices of (002), (020), (200), (120), (112), (022), (202), (222), (004), (040), (114), (024), (204), (124), and (204) accordingly. The peak values have indexes defined with monoclinic structures along with a space group P21/n as well as the lattice constant a = 7.3271, b = 7.5644, c = 7.7274 which makes them consistent with the standard JCPDS card No 43-1035 ^38^. The precisely defined and distinct intensity peaks indicate the formation of the WO_3_ crystallite nanoparticles, which did not contain any impurities, and no indication of secondary phase formation was detected, implying that Cu^2+^ ions have been effectively incorporated into the WO_3_ lattice.

**Fig. 4:**
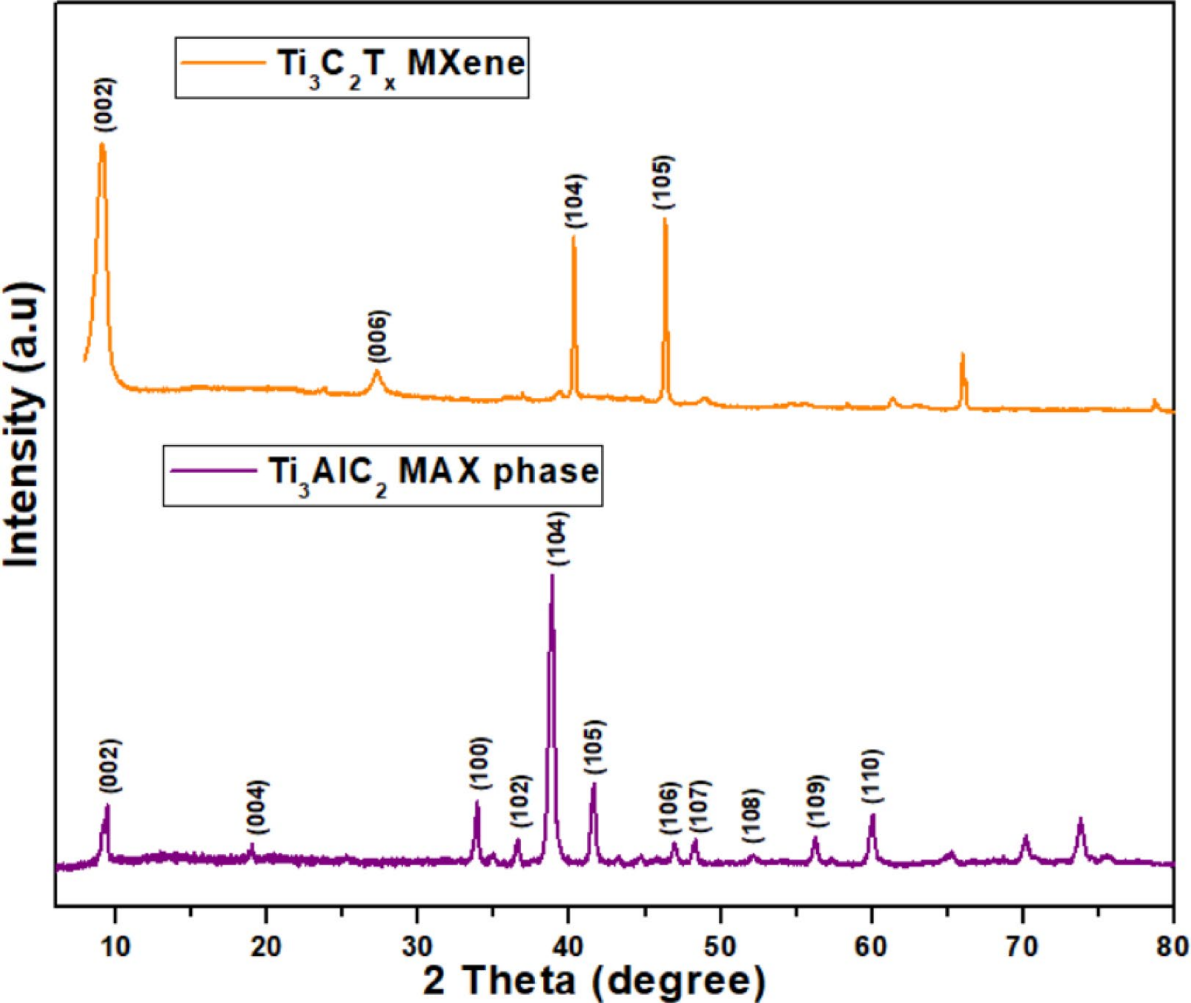
XRD spectra of MAX and Mxene.

The observed PXRD spectrum of Cu-doped WO_3_ nanoparticles exhibits increased intensity in contrast to pristine WO_3_ nanoparticles, which is apparent in Fig. 3. According to this, doping is extensively effective in stimulating crystallite formation and, consequently, facilitating the development of bonds between copper, tungsten, and oxygen. Figure 4 shows the XRD spectrum of the MAX phase (Ti_3_AlC_2_) and MXene (Ti_3_C_2_Tx). The XRD pattern of the pure MAX phase showed two theta peak locations at 9.5, 19.0, 33.8, 36.7, 39.0, 41.5, 46.9, 48.3, 52.2, 56.3, and 60.1. These locations correlate with the Miller indices (002), (004), (100), (102), (104), (105), (106), (107), (108), (109), and (110), respectively. In order to produce high-quality MXene, Al was substantially etched using HF. MXene’s distinctive peak can be observed at 2θ = 6. Additionally, a peak shift has also been detected at the peak. The following signifies that pure MXene has been effectively synthesized. All the distinctive peaks of WO_3_ and MXene can be observed in the WO_3_/MXene composite’s XRD pattern, indicating the composite’s successful preparation^39^.

**Fig. 5:**
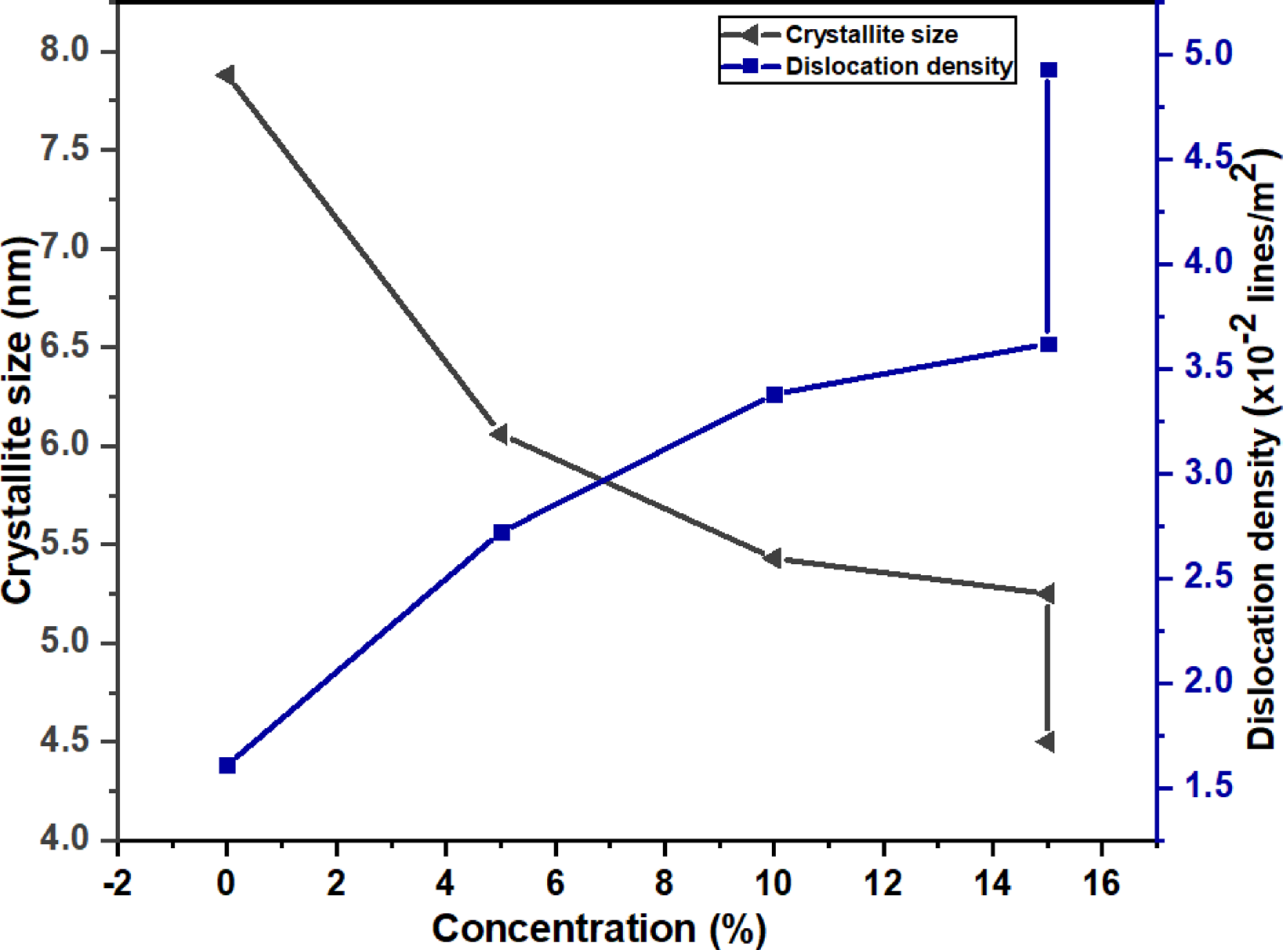
Variation of crystallite size and dislocation density of WO_3_, Cu-WO_3_ (Cu x% =5 at%, 10 at%, 15 at%) and 15 at% Cu-WO_3_/MXene nanocomposite.

The average size of the crystallite of the as-synthesized nanoparticles was measured by deploying the Debye-Scherrer equation described below.

1 $$\:\text{D}=\frac{\text{n}{\uplambda\:}}{{\upbeta\:}\text{cos}{\uptheta\:}}.$$

Wherein, D signifies the average crystallite size, n represents the Scherrer constant, which is 0.9, λ indicates the Cu Kα wavelengths, which is 1.5405 Å, and β and θ denote the full-width half maxima (FWHM) as well as the angle of the diffraction peaks^40^. Furthermore, some additional parameters derived from the XRD analysis include dislocation density (δ), micro-strain (ε), interplanar distance (d), lattice constants, and stacking fault (α). These values have been calculated and summarized in Table 1, and Fig. 5 shows the variation of crystallite size and dislocation density of WO_3_, Cu-WO_3_ (Cu x% =5 at%, 10 at%, 15 at%), and 15 at% Cu-WO_3_/MXene nanocomposite.

2 $$Interplanar \;distance \;d \;=\:\frac{1}{{\text{d}}^{2}}=\frac{1}{{\text{sin}}^{2}{\upbeta\:}}\left(\frac{{\text{h}}^{2}}{{\text{a}}^{2}}+\frac{{\text{k}}^{2}{\text{sin}}^{2}{\upbeta\:}}{{\text{b}}^{2}}+\frac{{\text{l}}^{2}}{{\text{c}}^{2}}-2\frac{\text{h}\text{l}\text{cos}{\upbeta\:}}{\text{a}\text{c}}\right)$$

3 $$\:Volume \;of \;unit \;cell \;V=abc \;Sin {\beta\:}$$

4 $$\:\text{D}\text{i}\text{s}\text{l}\text{o}\text{c}\text{a}\text{t}\text{i}\text{o}\text{n}\:\text{d}\text{e}\text{n}\text{s}\text{i}\text{t}\text{y}\:{\updelta\:}\:=\:\frac{1}{{\text{D}}^{2}}$$

5 $$\:\text{M}\text{i}\text{c}\text{r}\text{o}-\text{s}\text{t}\text{r}\text{a}\text{i}\text{n}\:{\upepsilon\:}\:=\:\:\frac{{\upbeta\:}}{4\text{tan}{\uptheta\:}}$$

6 $$\:\text{S}\text{t}\text{a}\text{c}\text{k}\text{i}\text{n}\text{g}\:\text{f}\text{a}\text{u}\text{l}\text{t}\:{\upalpha\:}\:=\:\left[\frac{2{{\uppi\:}}^{2}}{45{\left(3\text{tan}{\uptheta\:}\right)}^{\raisebox{1ex}{$1$}\!\left/\:\!\raisebox{-1ex}{$2$}\right.}}\right]{\upbeta\:}$$

**Table 1:** Parameters extracted from XRD spectra of Cu-WO_3_ and Cu-WO_3_/MXene nanocomposite (Fig. 5).

Parameters	WO_3_ (0 at%)	Cu-WO_3_ (5 at%)	Cu-WO_3_ (10 at%)	Cu-WO_3_ (15 at%)	CuWO_3_/MXene (15 at%)
Crystallite size ‘D’ (nm)	7.88	6.06	5.43	5.25	4.50
Dislocation density ‘$\:\varvec{\delta\:}\varvec{{\prime\:}}$ (x10^− 2^ lines/m^2)^	1.61	2.72	3.38	3.62	4.93
Micro-strain ‘$\:\varvec{\epsilon\:}\varvec{{\prime\:}}\:$(x10^− 1^) (Lines^− 2^m^− 4^)	2.33	3.03	3.36	3.50	4.07
stacking fault ‘$\:\varvec{\alpha\:}\varvec{{\prime\:}}$ (x10^− 1^)	1.09	1.42	1.58	1.64	1.91

### Fourier-transform infrared (FTIR) spectroscopy

**Fig. 6:**
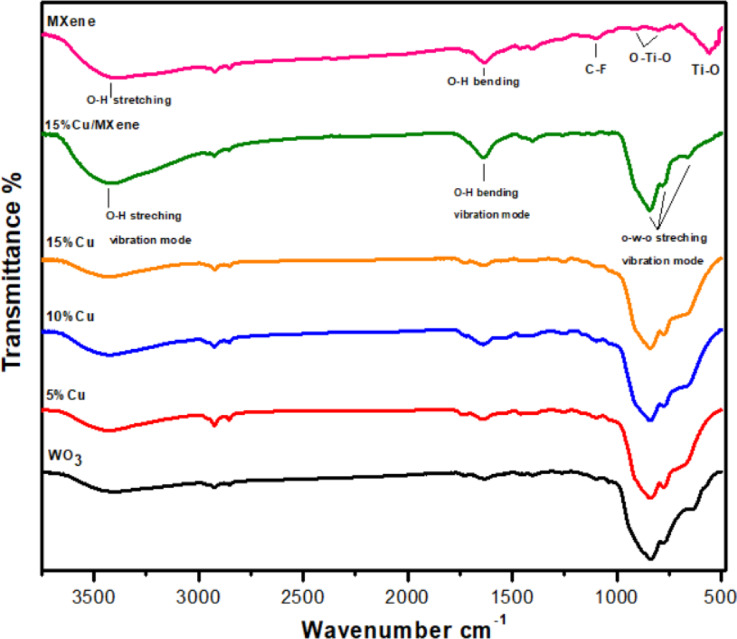
FTIR spectrum of MXene, WO_3_, Cu-WO_3_ (Cu x% = 5%, 10%, 15%) and 15% Cu-WO_3_/MXene nanocomposite.

The Fourier transform infrared spectroscopy (FTIR) is a significant chemical analysis technique that has been carried out at an ambient temperature, which offers crucial insights regarding both the compositional characteristics as well as surface functionalization of the synthesized materials, which are illustrated in Fig. 6. In the spectrum of FTIR, infrared (IR) light has been absorbed by molecules that contain functional groups with strong dipoles that exhibit prominent peaks. Figure 6 displays the FT-IR patterns of the MXene, WO_3_, Cu-WO_3_, and Cu-WO_3_/MXene nanocomposite that were measured in the range of 400–4000 cm^− 1^. The recorded spectra’s observed wavenumbers as well as their correlating assignments are listed in Table 2^41^. The monoclinic crystalline structure and single-phase purity of the pristine and Cu-doped WO_3_ nanoparticles have been confirmed through the detected FTIR bands. Analysis of the MXene (Ti_3_C_2_Tx) FTIR spectra reveals a number of distinctive bands that demonstrate substantial surface variations after etching. The stretching vibrations of –OH molecules are liable for the band at 3414 cm^− 1^, also the bending vibrations of hydroxyl (–OH) groups tend to be responsible for the band at 1630 cm^− 1^, underscoring the hydrophilic characteristics of the MXene surfaces. Furthermore, the bending vibrations of –OH molecules indicated by the absorption peak at 1630 cm^− 1^ show that water has adsorbed on the MXene surfaces. In addition, the narrow band at 554 cm^− 1^ is associated with the titanium atoms’ in-plane vibrations resulting from Ti-O bonding. Likewise, the apparent presence of C-F functionalities can be witnessed by the band at 1094 cm^− 1^, underscoring the inclusion of functional groups during the synthesis procedure^42^. The O-W-O stretching vibration is delineated by a wide band located at 550–1050 cm^− 1^. In cases where Cu is incorporated into WO_3_, the infrared band widens substantially, indicating that Cu has been efficiently doped into the main lattice of WO_3_. Additionally, the existence of various molecules or functional groups, such as water, may also be observed. The further vibration mode identified at 1635 cm^− 1^ tends to be bending vibrations by the hydroxyl (-OH) bond, resulting from the absorption/adsorption of water by the hydroxyl group. There exists another band around 3441 cm^−1^, which is associated with the stretching modes of the O-H groups in hydroxyls or water. The non-existence of Cu modes or other deliberately impurity-related modes in the FTIR spectrum affirms the successful insertion of the copper ions into the tungsten oxide parent material, thus ensuring the doping process. The Cu-WO_3_-doped MXene’s FTIR spectrum showed bands that were suggestive of several interactions involving titanium and tungsten compounds^43^. In particular, WO-O, Ti-O, TiO_2_, and Ti-C vibrations are represented by the absorption bands at 662 cm^− 1^, 554 cm^− 1^, 792 cm^− 1^, and 1094 cm^− 1^, respectively. These peak positions reflect how effectively tungsten oxide (WO_3_) has been incorporated into the MXene structure, which has been imperative in enhancing the material’s electrochemical characteristics. Furthermore, the results presented here disclose the existence of organic residues as well as surface modifications, which might optimize MXenes’ efficiency in electrochemical applications, emphasizing their use for a wide range of applications in battery storage and conversion technologies. As a whole, the FTIR analysis provides a thorough grasp of the surface chemistry of the synthesized materials while highlighting their structural integrity and functional versatility. The corresponding bond occurrence and peak positions are provided in Table 2.

**Table 2:** Vibrational band assignments for all absorption peaks of pure WO_3_, Cu-WO_3_ (Cu x% = 5%, 10%, 15%) and 15% Cu-WO_3_/MXene nanocomposite.

Assignment	WO_3_	Cu-WO_3_ (5 at%)	Cu-WO_3_ (10 at%)	Cu-WO_3_ (15 at%)	CuWO_3_/MXene (15 at%)
W–O bending mode	632.00	659.62	662.42	668.01	662.44
W–O–W stretching mode	771.06	771.06	771.47	771.47	775.95
W–O stretching mode	835.82	838.40	846.27	835.82	835.88
O-H bending vibration mode	1630.34	1635.91	1635.96	1630.34	1635.91
O-H stretching vibration mode	3431.07	3432.00	3432.19	3432.19	3441.11

### FESEM and EDS analysis

**Fig. 7:**
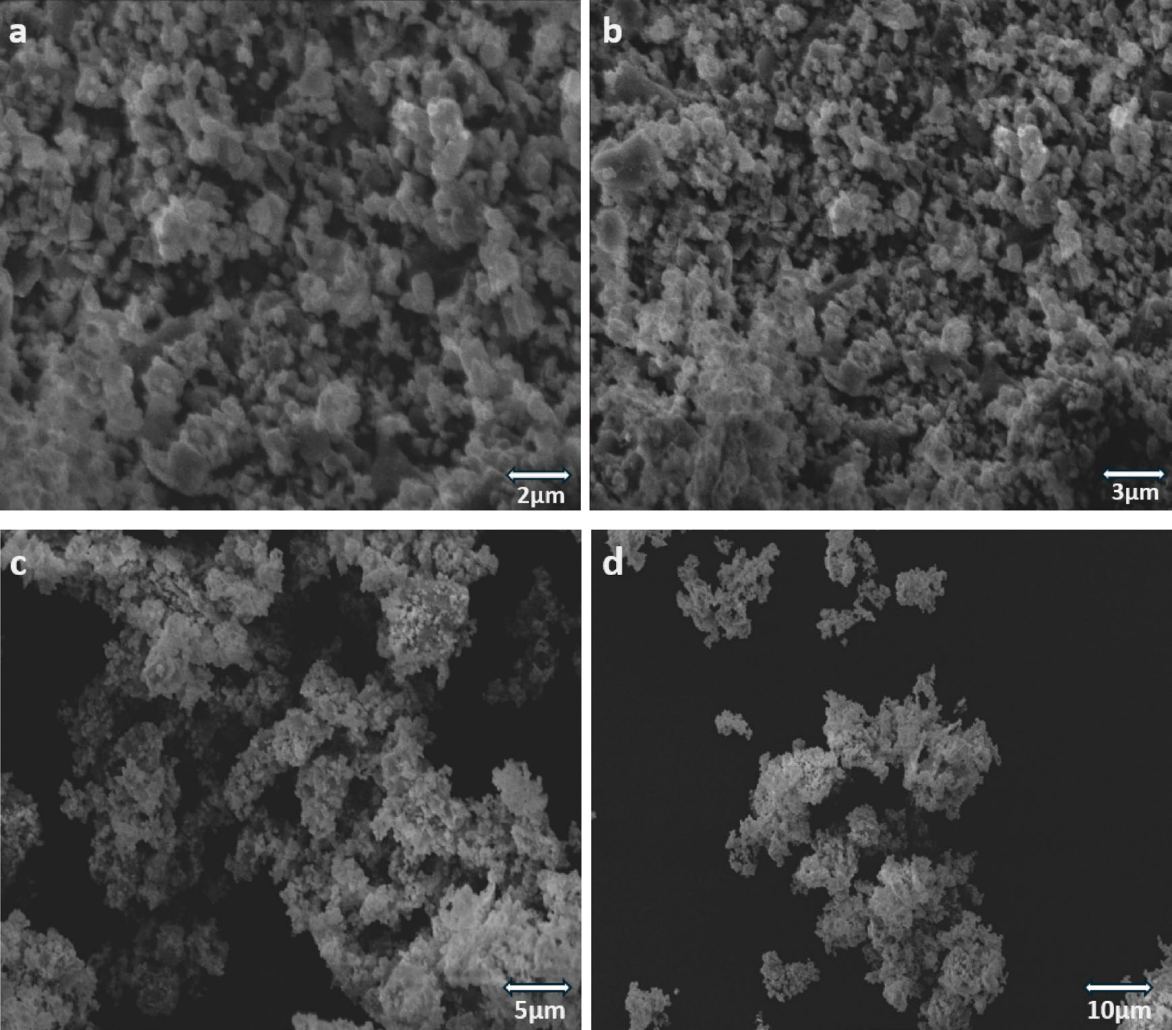

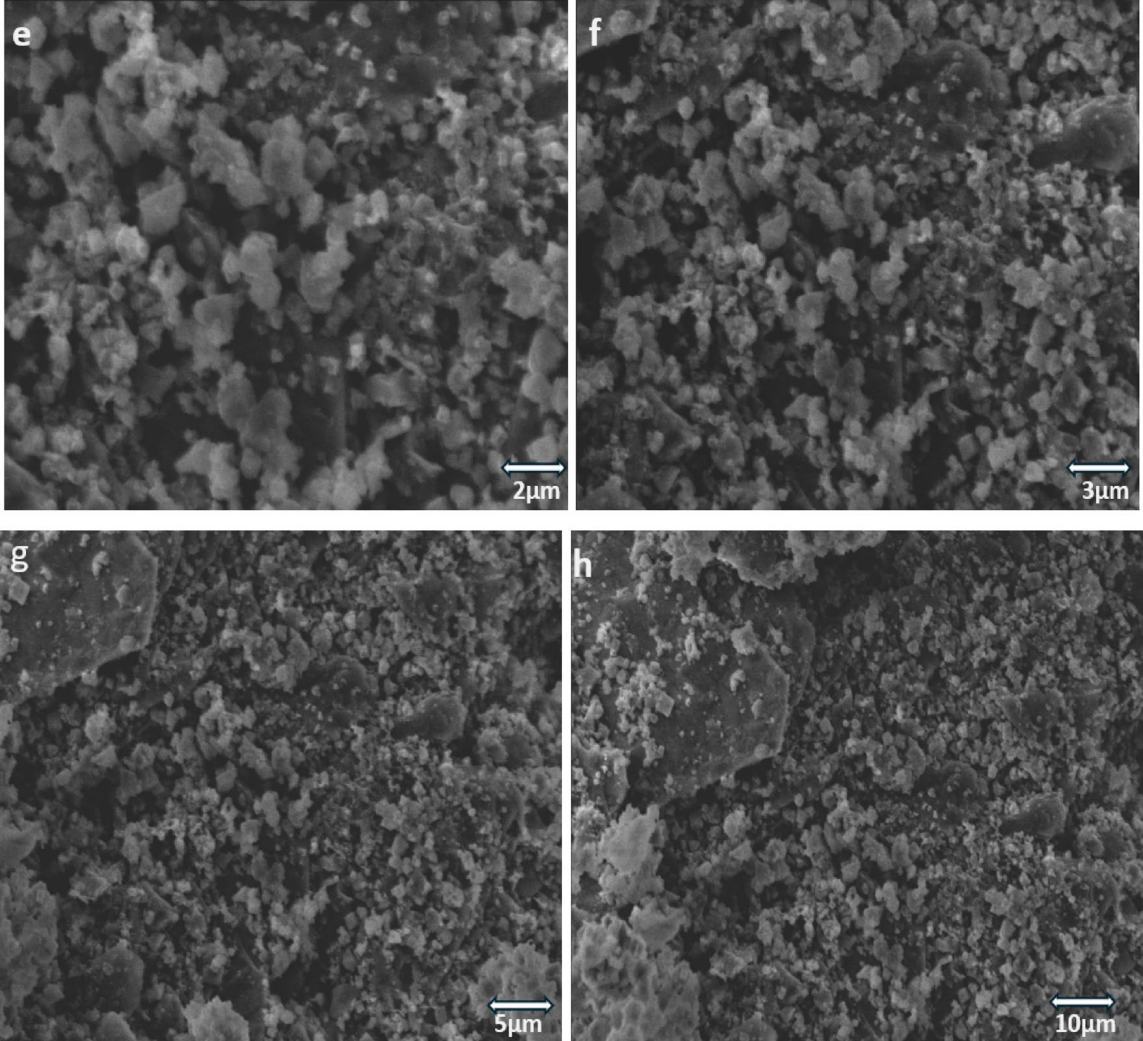
(**a**,** b**,**c** &** d**) FESEM images of 15 at% copper-doped tungsten oxide (Cu-WO_3_); (**e**,** f**,**g** &** h**) FESEM images of 15 at% Cu-WO_3_/MXene nanocomposite.

**Fig. 8:**
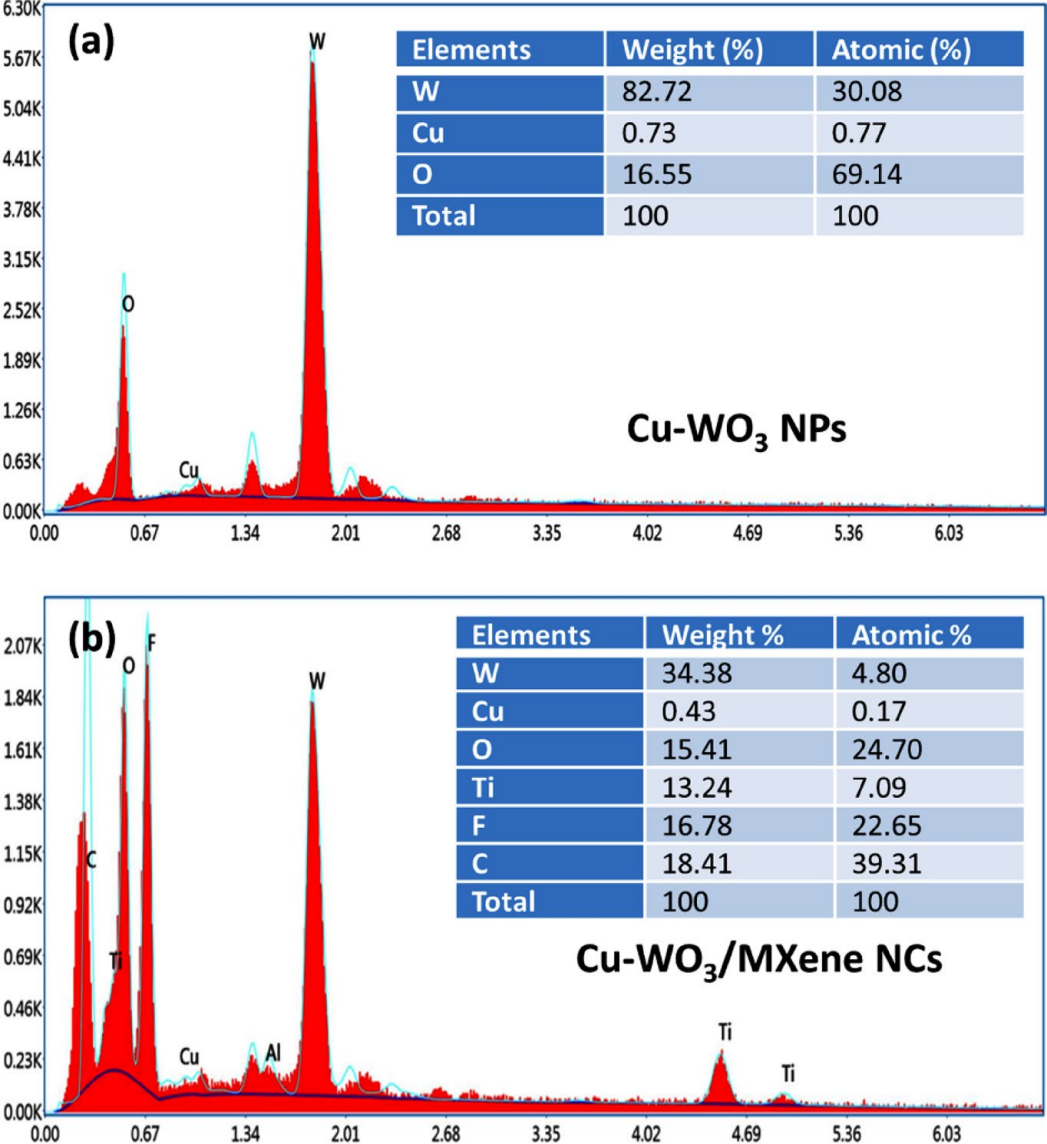
EDS pattern of (a) Cu-WO_3_ NPs, (b) Cu-WO_3_/MXene NCs.

Field-emission scanning electron microscopy (FESEM) (Fig. 7) and energy dispersive spectroscopy (EDS) (Fig. 8) analysis were performed to investigate the surface morphological characteristics and elemental composition of the as-prepared Cu-WO_3_ and 15 at% Cu-WO_3_/MXene nanocomposites. Low-scale micrographic imaging of the nanostructures under various magnifications is displayed in Figs. 7(a–h). The images obtained from the SEM (Figs. 7(a-d)) disclose the formation of clusters with sphere-like morphology for Cu-doped WO_3_ particles. The imagery exemplifies the presence of intensely agglomerated nanoparticles. The particle agglomeration and size variations are the outcome of the coprecipitation technique, which aggregates the particles through multiple simultaneous processes, leading to an ample variety of particle sizes. The spheres of Cu-doped tungsten oxide nanoparticles can be seen embedded upon MXene sheets in the Cu-WO_3_/MXene nanocomposite SEM images (Figs. 7(e–h)). Effective embedding of Cu-WO_3_ nanoparticles leads to their segregation from agglomerated conditions, resulting in individual spheres upon MXene sheets. Also, the MXene fabrication is represented by the nanosheet-like structure in the preceding image. The Cu-doped WO_3_ nanoparticles placed between MXene layers prevent them from reassembling^44^. As a result, it increases surface area and renders efficient ways for enhancing its electrochemical characteristics.

Energy dispersive spectroscopy (EDS) has been employed to ascertain both the elemental composition as well as the purity of the produced electrode samples. The EDS spectra of the fabricated Cu-WO_3_ and Cu-WO_3_/MXene nanocomposite are illustrated in Figs. 8(a, b). It is evident that the EDS spectra clearly demonstrate that the created 15 at% copper-doped tungsten oxide comprises W, Cu, and O components, without any additional elements present, confirming the as-synthesized sample’s purity. Furthermore, the elemental mapping of the material has been performed in order to observe how the elements were distributed. The results obtained are displayed in Fig. 8(a), thereby confirming that elements such as W, O, and Cu are dispersed consistently throughout the region and also proving that the copper ions are effectively doped in WO_3_. The results obtained are displayed as shown in Fig. 8**(a)**, thereby confirming the elements such as W, O, and Cu are dispersed consistently throughout the region and also proving that the copper ions are effectively doped in WO_3_. Figure 8**(b)** depicts the EDS pattern for the Cu-WO_3_/MXene nanocomposite, revealing the inclusion of W, Cu, O, Ti, C, and F elements. Aside from these elements, no additional elements have been detected that validate the material’s purity. The addition of MXene results in the presence of Ti and C components. All of the aforementioned elements are evenly distributed over the specified region, as shown by the mapped area in Fig. 8(b)^45^. The as-synthesized 15 at% Cu-WO_3_ and Cu-WO_3_/MXene nanocomposites’ compositional percentages (weight and atomic percentages) are listed in the inset table of Fig. 8.

HRTEM analysis

**Fig. 9:**
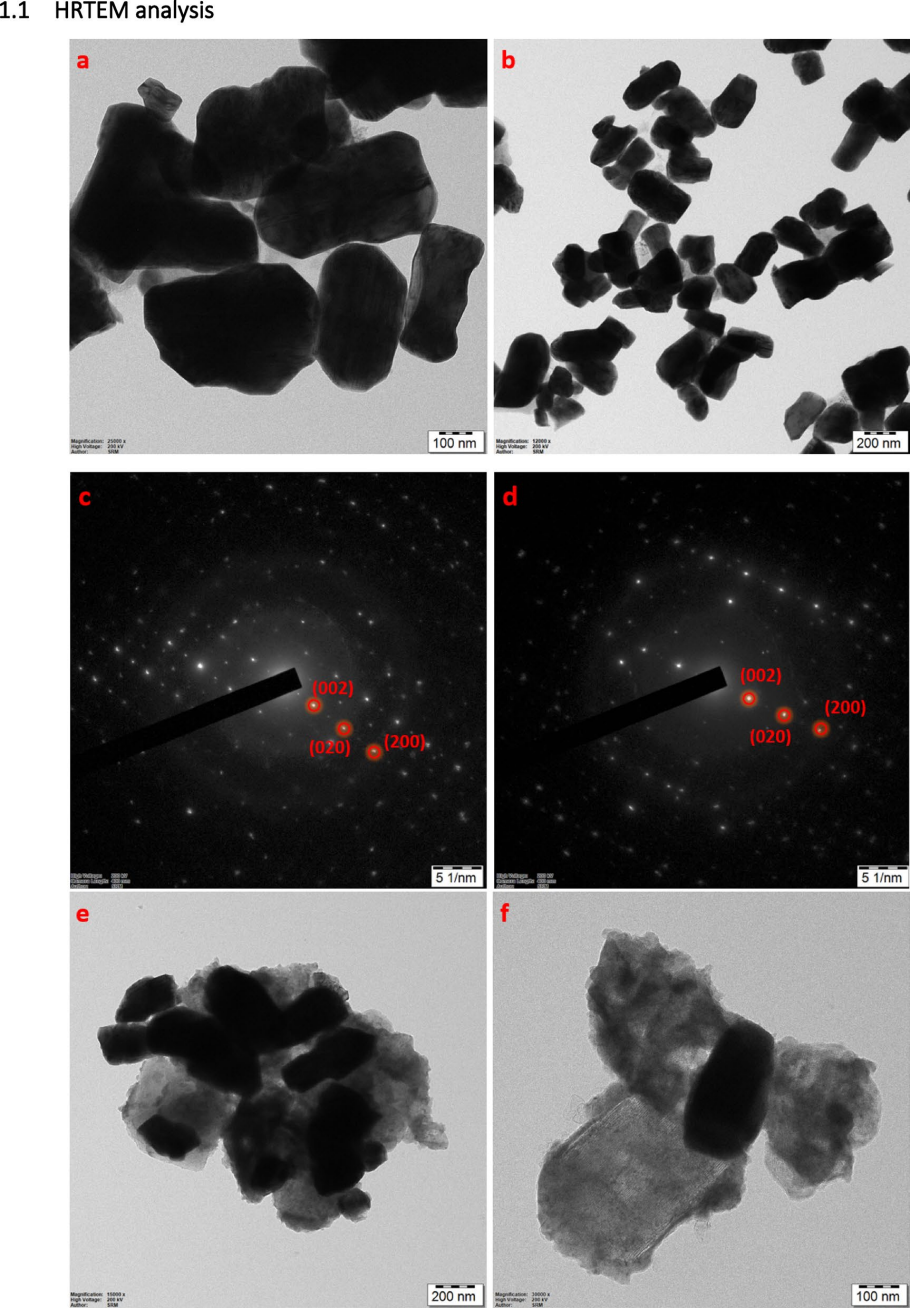

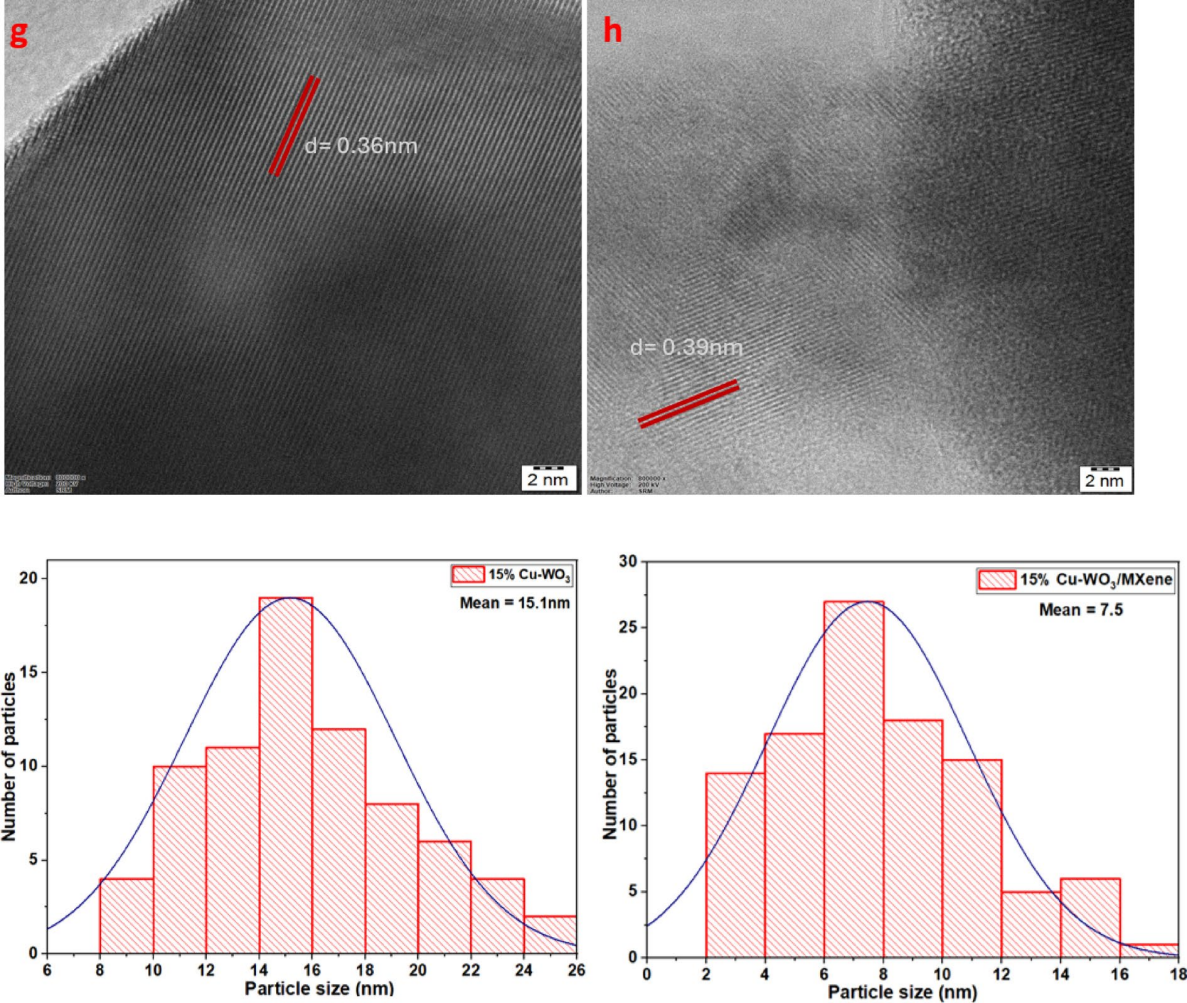
(a&b) HRTEM images of 15 at% copper-doped tungsten oxide NPs (Cu-WO_3_), (c&d) SAED pattern of 15 at% Cu-WO_3_ NPs and Cu-WO_3_/MXene NCs, (e&f) HRTEM images of Cu-WO_3_/MXene NCs, (g&h) lattice fringes of 15 at% Cu-WO_3_ NPs and Cu-WO_3_/MXene NCs, (i&j) Histogram showing the particle size distribution of 15 at% Cu-WO_3_ NPs and Cu-WO_3_/MXene NCs.

The HRTEM analyses were carried out in order to examine the core level morphology as well as to correlate the crystallinity of the Cu-WO_3_ and 15 at% Cu-WO_3_/MXene nanocomposites, as shown in Figs. 9 (a-h). Higher magnifications of Figs. 9(a&b) show the uniform development and interconnected nanorod-like networks. Additionally, as can be seen from the magnified imagery in Figs. 9(e&f), Cu-WO_3_ nanorods have been embedded in the Ti_3_C_2_T_x_ MXene sheets. An increasing concentration of Cu-WO_3_ nanorods is discernibly observed in the Ti_3_C_2_ MXene grain boundaries in Figs. 9(e–f), indicating that Cu-WO_3_ is efficiently deposited and substantially covers the entire surface throughout the bath sonication process. Furthermore, the crystals’ interlayer spacing (d spacing) is 0.36 nm for Cu-WO_3_ and 0.39 nm for Cu-WO_3_/MXene nanocomposite (illustrated in Figs. 9(g&h))^46^. The interplanar spacing analysis from HRTEM reveals a distinct difference between the two samples. For 15 at% Cu-doped WO_3_, a lattice fringe spacing of approximately 0.36 nm was observed, corresponding to the (200) plane of the monoclinic WO_3_ phase. Upon integration with MXene, the interlayer spacing increased to approximately 0.39 nm, indicating a slight lattice expansion. This expansion suggests possible intercalation or structural distortion induced by the incorporation of Cu-WO_3_ into the MXene layers. Such a shift implies enhanced interaction at the interface between the two materials, which can benefit electrochemical performance by providing more open ion diffusion channels. This observation is supported by XRD analysis, where a slight shift of the (200) diffraction peak toward lower 2θ values was noted in the Cu-WO_3_/MXene composite compared to pristine Cu-WO_3_. This shift confirms the lattice expansion observed in HRTEM, further suggesting successful integration and structural modification of the composite system. Moreover, the nanorod clusters in the composite predominantly grow along the (200) crystallographic direction, maintaining the monoclinic WO_3_ phase. These uniformly oriented nanorods on the MXene surface form efficient ion transport pathways, facilitating electrolyte penetration and promoting faster electrochemical reactions. The synergistic effect of copper doping and MXene integration potentially enhances the faradaic activity and charge transport kinetics, making the material highly suitable for advanced energy storage applications^47,48^. Furthermore, a distinct diffraction ring pattern is visible in the selected area of electron diffraction (SAED) illustrated in Figs. 9(c&d), where the spots indicate the polycrystalline nature of the synthesized material^49^. These rings have been indexed based on the standard JCPDS card for monoclinic WO_3_ (JCPDS No 43-1035), and the corresponding lattice planes, such as (002), (020), and (200), are clearly marked in the SAED images. The observed diffraction features are consistent with the crystallographic structure of Cu-doped WO_3_, indicating that the incorporation of copper and MXene does not disrupt the primary monoclinic phase. In the composite sample, additional faint rings associated with the MXene phase may also be observed, suggesting the successful integration of the two components.

### XPS analysis

**Fig. 10:**
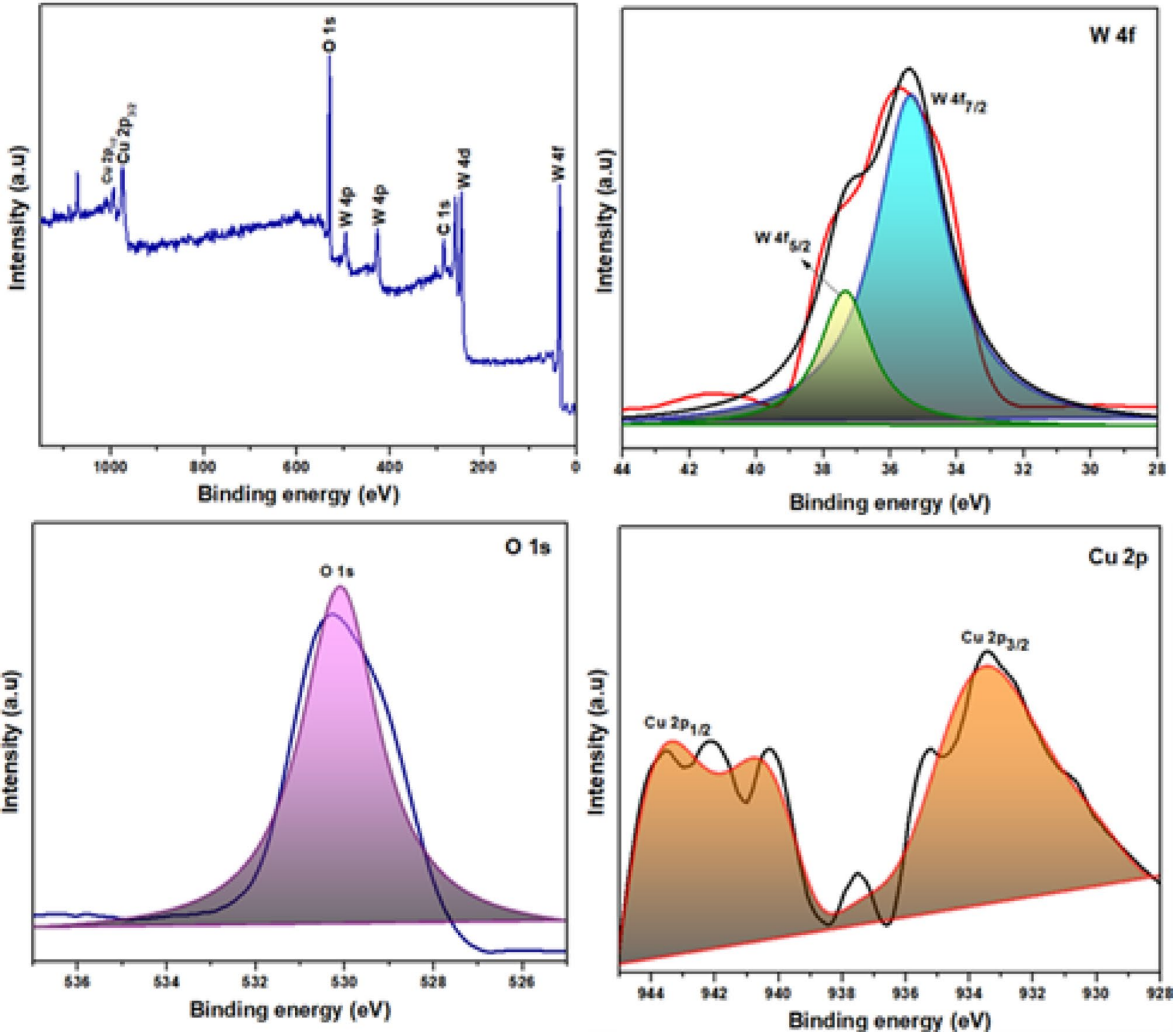
XPS spectra of Cu-WO_3_ NPs.

**Fig. 11:**
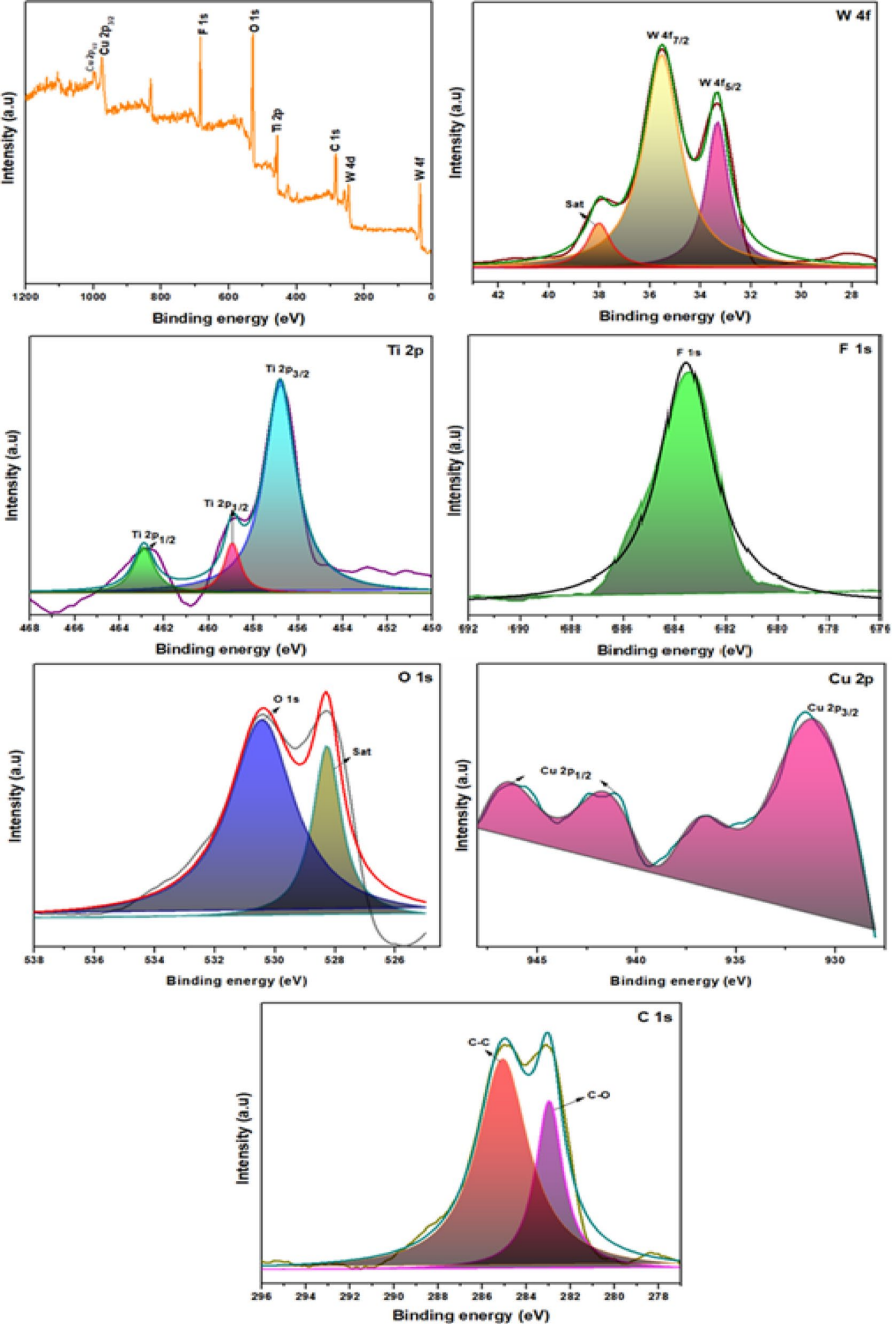
XPS spectra of Cu-WO_3_/MXene composite.

In order to examine the chemical composition of the surface, binding energies, and oxidation states of different elements present in as-synthesized Cu-WO_3_ and Cu-WO_3_/MXene nanocomposites, X-ray photoelectron spectroscopy (XPS) has been performed. Figures 10 and 11 display the results of the XPS spectra revealing the element peaks of tungsten (W 4f), copper (Cu 2p), oxygen (O 1s), carbon (C 1s), titanium (Ti 2p), and fluorine (F 1s). These high-resolution XPS peaks are deconvoluted utilizing the Voigt function fitting with Shirley background subtraction^50^. The Cu-WO_3_ spectra (see Fig. 10) exhibit prominent peaks corresponding to W, Cu, and O, confirming the presence of these elements. These peaks exhibit distinct binding energy patterns, as corroborated by XPS results. However, the elements W, O, and Cu photoelectron peaks in (Fig. 10) are attributed to W 4f, O 1s, and Cu 2p. Two photoelectron peaks corresponding to W 4f 5/2 and W 4f 7/2 are apparent in (Fig. 10) at energies of about 37.41 and 35.33 eV, as well as 35.52 and 33.25 eV, respectively, in Cu-WO_3_ and 15 at% Cu-WO_3_/MXene nanocomposite^51^. The aforementioned peaks are correlated with the exceptionally defined W 4f band, which contains a spin-orbit doublet representing the W valence phase. The spin-orbit split takes place in the f-core stage, and it also shows that the W element is at + 6 valence. The above-mentioned values confirm that there has been a slight shift observed in MXene-doped samples in comparison to the pristine. A single O 1s photoelectron peak is evident in Fig. 10 at 530.08 eV for Cu-WO_3_ and at 530.41 eV for the 15 at% Cu-WO_3_/MXene nanocomposite. These are associated with the W–O bond and demonstrate that the electrode material encompasses metallic oxygen. The four peaks or doublets of Cu 2p in Figs. 10 and 11 correspond to Cu 2p3/2 and Cu 2p1/2 and appear after the curve has been deconvoluted. They are located at energies of about 933.44, 942.18 eV, and 932.18, 943.44 eV, respectively. These findings validate the Cu-WO_3_ phase and demonstrate that Cu has been effectively incorporated into WO_3_. The Cu-WO_3_/MXene composite’s survey spectrum, illustrated in Fig. 11, verifies the existence of the elements W, Cu, O, F, C, and Ti^52^. The fitted C 1s spectra display two prominent peak values at 285.19 and 282.91 eV, as illustrated in Fig. 11. These peaks are ascribed to C-C and C-O, respectively. The titanium 2p spectra of the composite can be divided into three distinct peaks, as seen in Fig. 11. Two of these peaks, situated at 462.6 and 458.5 eV, are ascribed to the Ti 2p 3/2 and Ti 2p 1/2 bonds in the composite material, accordingly. Likewise, the energy peak at 456.1 eV has been attributed to the Ti-C bond. The observed peak at 462.6 eV is attributable to the Ti-F interaction. Similarly, the peak value at 458.5 eV indicates the binding energy of the C-Ti-O, which represents the bond between surface groups in MXene. The results obtained clearly demonstrate that the surface termination groups, including hydroxyl (-OH) and fluoride (-F) groups, have substituted the aluminum (Al) layer in the MAX phase. A deconvoluted graph of the F1s spectra is apparent in Fig. 11, where the C-Ti-F strong peak is located at 683.52 eV, respectively. The slight shift in binding energies of W 4f, O 1s, and Cu 2p observed in the Cu-WO_3_/MXene nanocomposite, as compared to pristine Cu-WO_3_, provides evidence of a chemical interaction or electronic environment change due to the integration with MXene. This suggests a successful coupling between Cu-WO_3_ and the MXene substrate, potentially improving charge transfer and enhancing electrochemical performance. The appearance of Ti–C, Ti–O, and Ti–F bonds in the Ti 2p spectra of the composite further supports the interaction between MXene surface terminations and Cu-WO_3_ nanorods. These interactions can be attributed to interfacial bonding or partial charge redistribution at the Cu-WO_3_/MXene interface. These spectroscopic shifts and bond characteristics collectively confirm the successful integration and interfacial interaction between Cu-WO_3_ and MXene, as expected in hybrid nanostructures for improved supercapacitor applications^53^.

### Electrochemical investigations

#### Cyclic voltammetry analysis (CV)

**Fig. 12:**
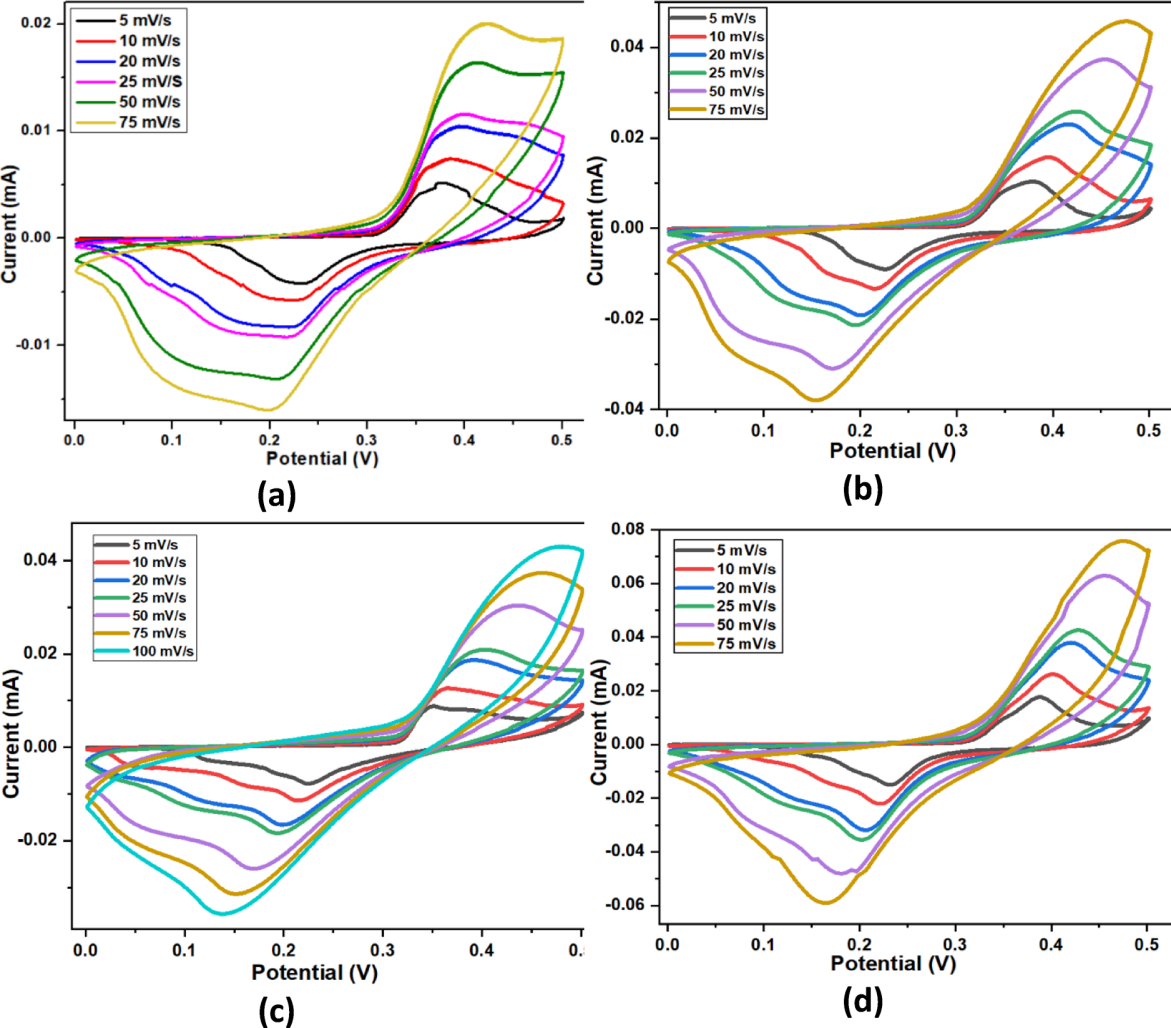

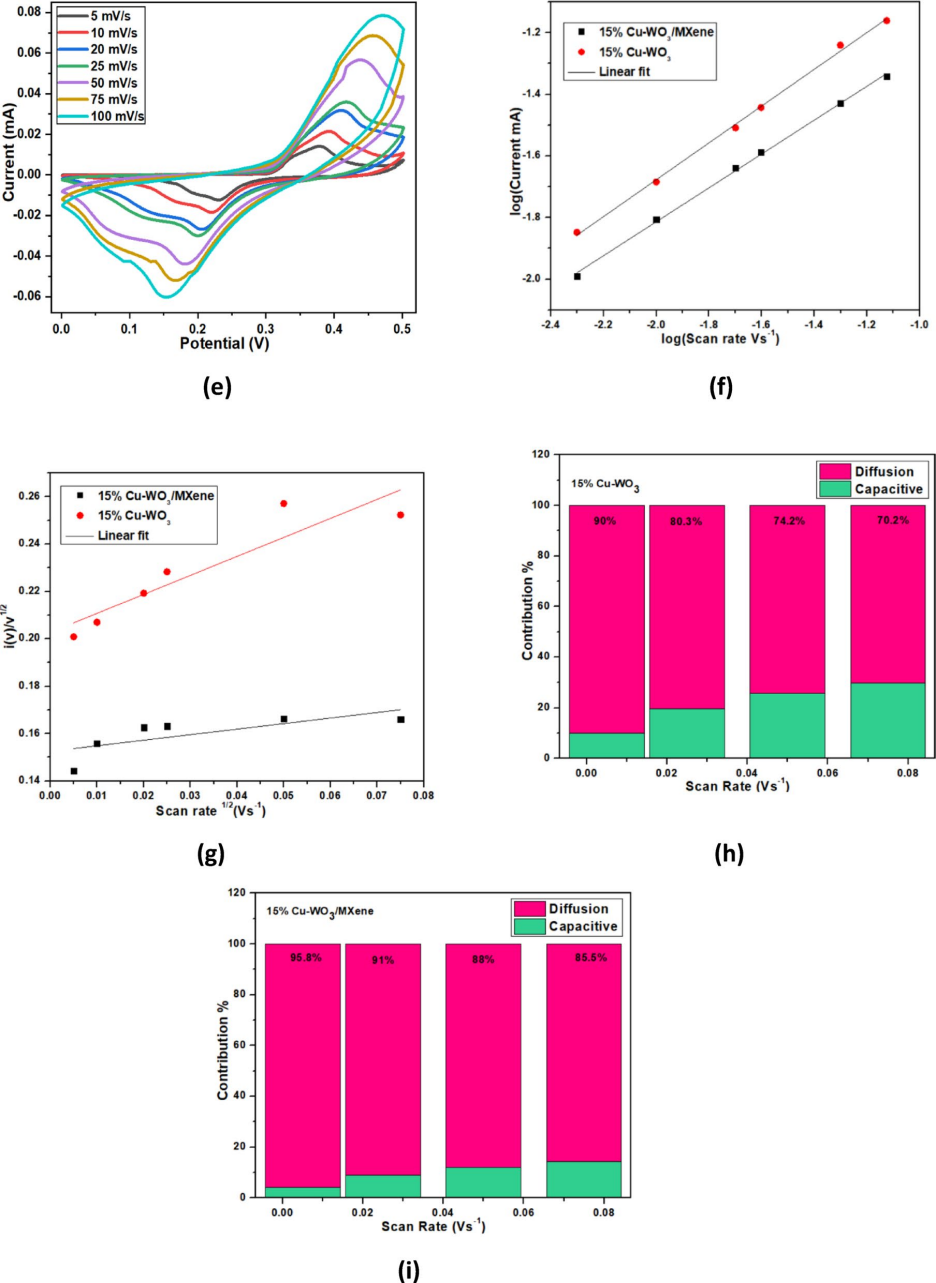
CV diagram of (**a**) WO_3_, (**b**) 5 at% Cu-WO_3_ (**c**) 10 at% Cu-WO_3_ (**d**) 15 at% Cu-WO_3_ (**e**) 15 at% Cu-WO_3_/MXene nanocomposites at various scan rates from 1 to 100 mV/s (**f**) linear plot of log(v) vs. log(i) (**g**) scan rate^1/2^vs. i(v)v^1/2^ (**h** &** i**) contribution percentages of capacitive and diffusive percentage in 15 at% Cu-WO_3_ and 15 at% Cu-WO_3_/MXene nanocomposites.

The electrochemical analysis was conducted using cyclic voltammetry (CV) in a 1 M potassium hydroxide (KOH) electrolyte solution, utilizing a configuration with three electrodes. Figures 12(a–e) presents the cyclic voltammetry profiles of the synthesized WO_3_, Cu-doped WO_3_ (5 at%, 10 at%, and 15 at%), and 15 at% Cu-WO_3_/MXene nanocomposites. The measurements were carried out in the potential range spanning 0 to 0.5 V, along with scan rates varying from 5 to 100 mV/s. Furthermore, the nanocomposites’ CV profile displays a well-defined redox peak in a nearly rectangular form, indicating pseudo-capacitance behavior that may be attributed to the faradaic redox reaction occurring in the KOH electrolyte. A comparative study of the CV graphs resulting from pure WO_3_, 5 at%, 10 at%, and 15 at% Cu-doped WO_3_, and 15 at% Cu-WO_3_/MXene nanocomposites is illustrated in Figs. 12(a–e). The prominent redox responses observed at varying sweep rates confirm the electrochemical activity of the nanocomposites^29^. Throughout the insertion and extraction process, K^+^ ions engage in redox interactions with Cu^2+^ ions, facilitating the electrochemical mechanism by integrating Cu^2+^ into the lattice. It has been found that incorporating Cu as a grain boundary or substitutional, increases the specific capacitance value. Furthermore, the 15 at% Cu-WO_3_/MXene nanocomposites’ CV curves display a larger CV area, likely due to their large surface area and maximal peak current. Additionally, the redox peaks of these electrodes were more precisely defined than those of the pure WO_3_, 5 at%, 10 at%, and 15 at% Cu-doped WO_3_ electrodes, indicating that they had a greater capacity for charge storage kinetics and higher electrochemical reversibility. For 15 at% Cu-WO_3_/MXene nanocomposites, Fig. 12(e) exhibits a higher oxidation peak current and a slightly lower reduction peak current compared to other samples. This suggests enhanced redox activity and improved charge storage capability due to the synergistic effect of Cu doping and MXene incorporation. The increased oxidation peak current indicates more efficient faradaic reactions, while the slight reduction in cathodic current may be attributed to rapid charge transfer kinetics and improved electronic conductivity. During the cathodic scan, a small number of charges will be stored between the electrode material and electrolyte; these stored charges are then released during the anodic scan, resulting in distinct peaks. The specific capacitance (Cs) values of WO_3_, Cu-doped WO_3_ (Cu x% = 5 at%, 10 at%, 15 at%), and 15 at% Cu-WO_3_/MXene nanocomposites were computed using Eq. (7) and are listed in Table 3, where S refers to the surface area under the CV curve, m denotes the mass of the active material, Δv is the potential window, and k indicates the scan rate.

7 $$\:Cs=\frac{S}{mk\varDelta\:V} (F/g)$$

**Table 3:** Specific capacitance (C_s_) values were obtained from the CV curve.

Electrode Material	Scan Rate (mV/s)	Specific Capacitance ‘C_s_’(F/g)
WO_3_	5	204
	75	74.6
Cu-WO_3_ (5 at%)	5	380
	75	14.8
Cu-WO_3_ (10 at%)	5	380
	75	126.6
Cu-WO_3_ (15 at%)	5	520
	75	20
CuWO_3_/Mxene (15 at%)	5	660
	75	20

The mass loading of the active material on each electrode was approximately 2 mg, measured using a microbalance prior to electrochemical testing. At higher scan rates, ions in the electrolyte do not have sufficient time to penetrate deeper into the electrode material, resulting in a decreased specific capacitance. The shift in redox peaks indicates polarisation in conjunction with ion diffusion. The introduction of 15 at% Cu into the WO_3_ lattice and the subsequent formation of Cu-WO_3_/MXene nanocomposites lead to the formation of additional grain boundaries and active sites. These grain boundaries act as charge storage centers, facilitating ion diffusion and enhancing electrochemical performance. However, excessive grain boundary formation can sometimes hinder charge transport. In this case, the optimized Cu concentration balances the increase in active sites and conductivity, resulting in an improved specific capacitance. Thus, 15 at% Cu-doped WO_3_/MXene yields the highest specific capacitance value, around 660 F/g, suggesting that 15 at% Cu-WO_3_/MXene nanocomposites tend to be an excellent choice in tailoring the electrochemical characteristics^54^. Furthermore, to investigate the charge storage mechanism, CV curves were recorded at various scan rates ranging from 5 to 100 mV/s. The peak current (i) was plotted against the scan rate (v) in a log-log plot to obtain the b-value using the power law relationship i = a·v^b^, where i represents the peak current (A) and v is the scan rate (mV/s). The calculated b-value indicates that the charge storage process involves a combination of surface-controlled capacitive behavior and diffusion-controlled intercalation, as shown in Fig. 12(f–i).

#### Galvanostatic charge-discharge (GCD)

**Fig. 13:**
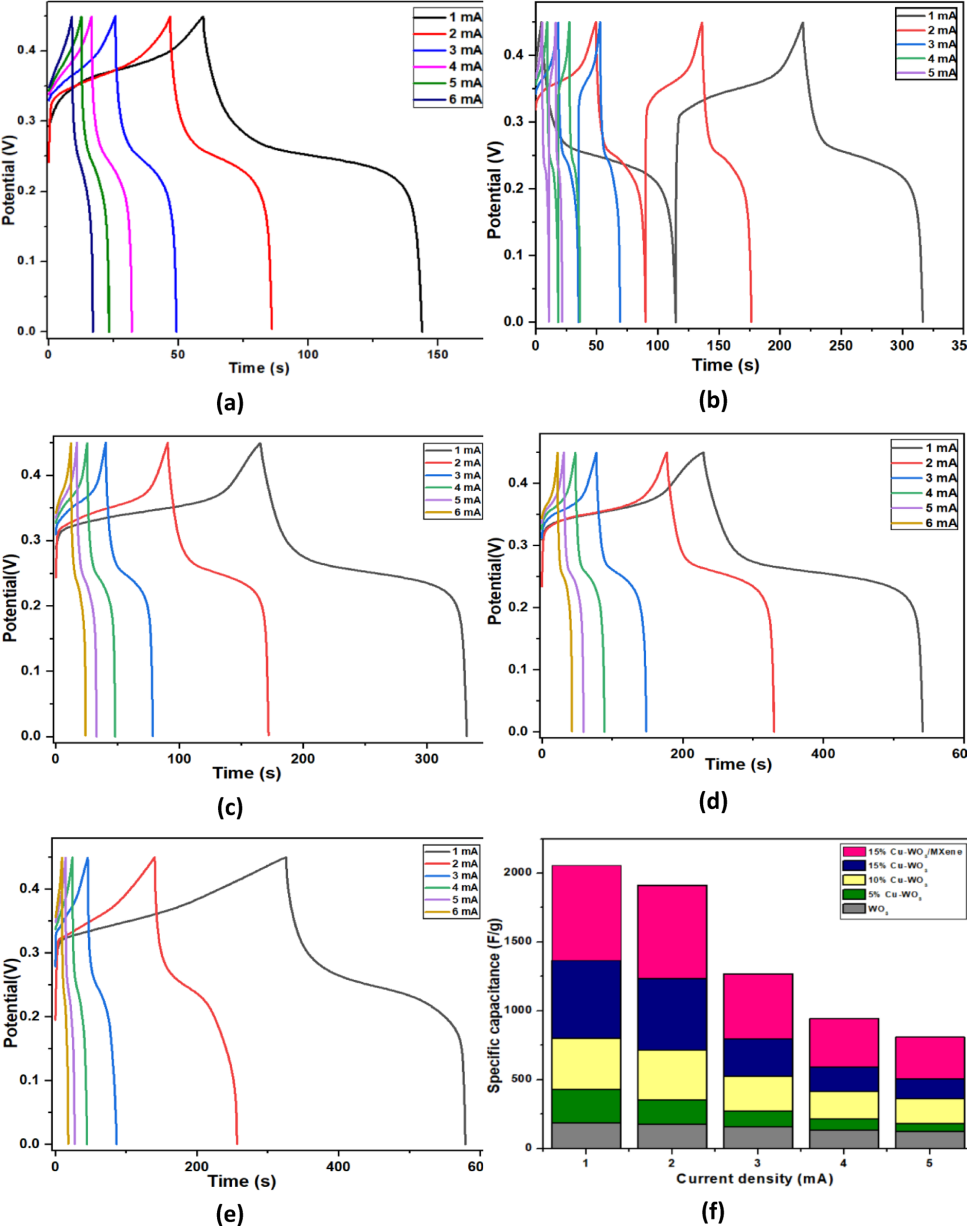
GCD curvatures of (a) WO_3_, (b) 5 at% Cu-WO_3_ (c) 10 at% Cu-WO_3_ (d) 15 at% Cu-WO_3_ (e) 15 at% Cu-WO_3_/MXene nanocomposites at multiple current densities (f) Specific capacitance with increasing current density.

The as-synthesized electrodes’ electrochemical performance was assessed using galvanostatic charge-discharge (GCD) testing, which was carried out between a potential window of 0 to 0.5 V. The GCD patterns of WO_3_, Cu-WO_3_ (Cu x% = 5 at%, 10 at%, and 15 at%), and 15 at% Cu-WO_3_/MXene electrode materials at various current densities are displayed in Figs. 13(a–e). Here, the extended discharging period amongst the nonlinear GCD profile suggested that the electrodes exhibited higher energy storage capacity^55^. GCD profiles for the aforementioned materials show significant discharge platforms, revealing a high pseudo-capacitance. The quasi-triangular GCD curve of Cu-WO_3_ and MXene nanocomposites indicates that redox processes at the electrode/electrolyte interface cause the composites to behave like pseudo-capacitors^56^. The respective electrode material’s specific capacitance is determined from the GCD curves using the given formula (Eq. 8).

8 $$\:{C}_{P}=\frac{I\varDelta\:t}{m\varDelta\:V} (F/g)$$

Where Cp signifies the specific capacitance (F/g), I indicates charging/discharging current (A), Δt is the discharge time, m is the active material mass, and ΔV indicates the potential difference of the curve. Evidently, the composite (15 at% Cu-WO_3_/MXene) electrodes exhibit higher specific capacitance and longer charge-discharge durations when compared to the pristine WO_3_ and Cu-WO_3_-based electrodes (Fig. 13(f)). The specific capacitance Cp of the electrodes was calculated from GCD plots at current densities of 1, 2, and 3 mA and has been tabulated^57^. Furthermore, as current density increases, it gets more difficult to inject K^+^ into the electrode, ultimately resulting in a decrease in the specific capacitance of equal electrode. At a lower current density, the insertion of K^+^ ions takes considerably more time to penetrate the electrode. In comparison to other trials, which included pristine WO_3_ and Cu-WO_3_ (Cu x% = 5 at%, 10 at%, and 15 at%) based electrode materials, the electrochemical characteristics of the 15 at% Cu-WO_3_/MXene nanocomposite were equivalent to or more efficient than those indicated in Table 4. The integration of MXene in 15 at% Cu-WO_3_/MXene nanocomposite increases the material’s discharge time period and shows that the specific capacitance increases, thereby enhancing its potential for high-performance super capacitive applications^58^. In addition, the energy density (E_D_)and power density (P_D_) of the samples have been determined and tabulated using the following formulas (Eqs. 9 and 10) (Table 5).

9 $$\:{E}_{D}=\frac{1}{2}{C}_{p}{\left(\varDelta\:V\right)}^{2} (WhKg ^{{ - 1}} )$$

10 $$\:{P}_{D}=\frac{ED\times\:3600}{\varDelta\:t}\:(WKg ^{{ - 1}} )$$

**Fig. 14:**
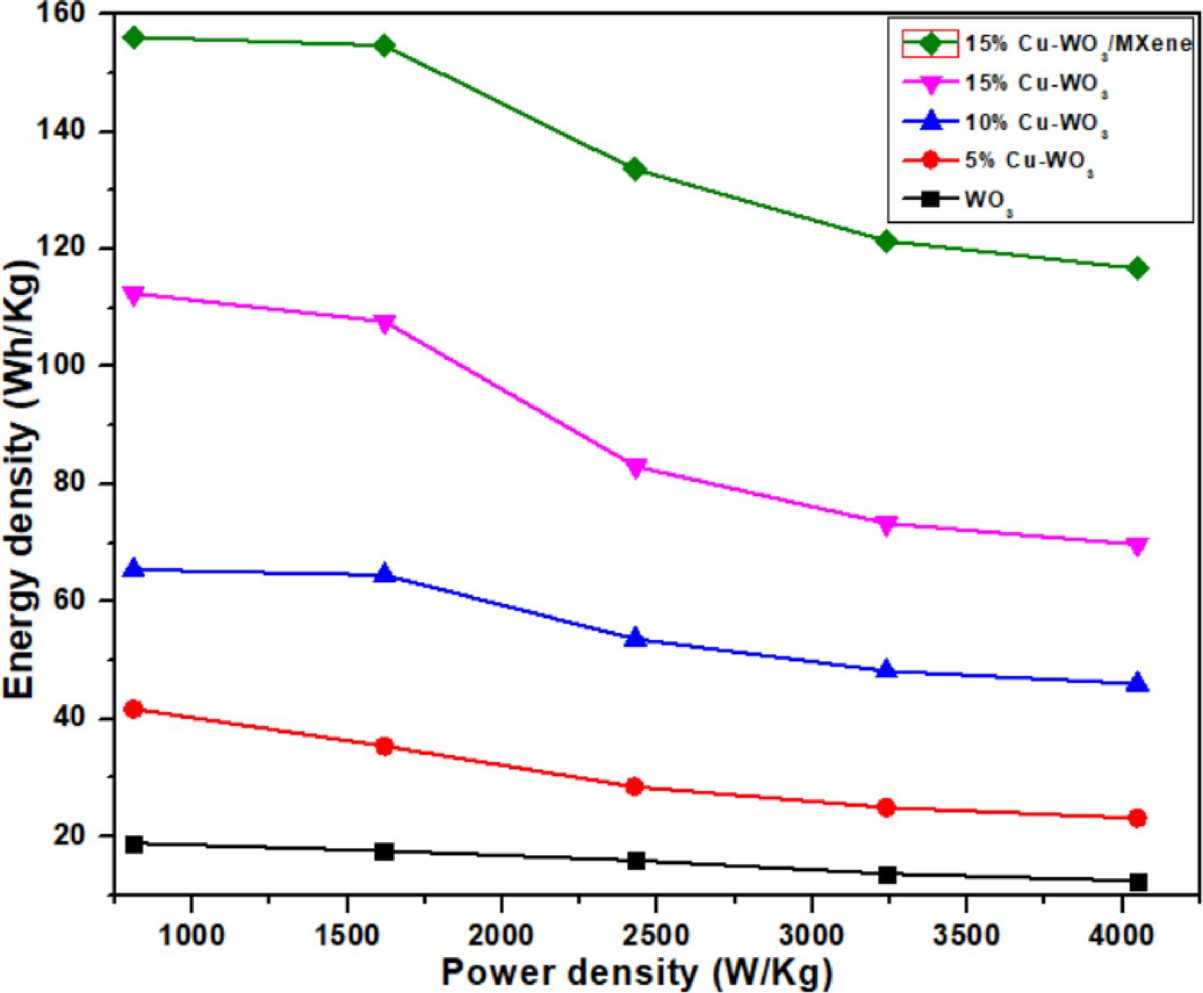
Ragone plots comparing the energy and power densities of WO_3_, Cu-WO_3_ (Cu x% = 5%, 10%, 15%), and 15% Cu-WO_3_/MXene nanocomposite.

**Fig. 15:**
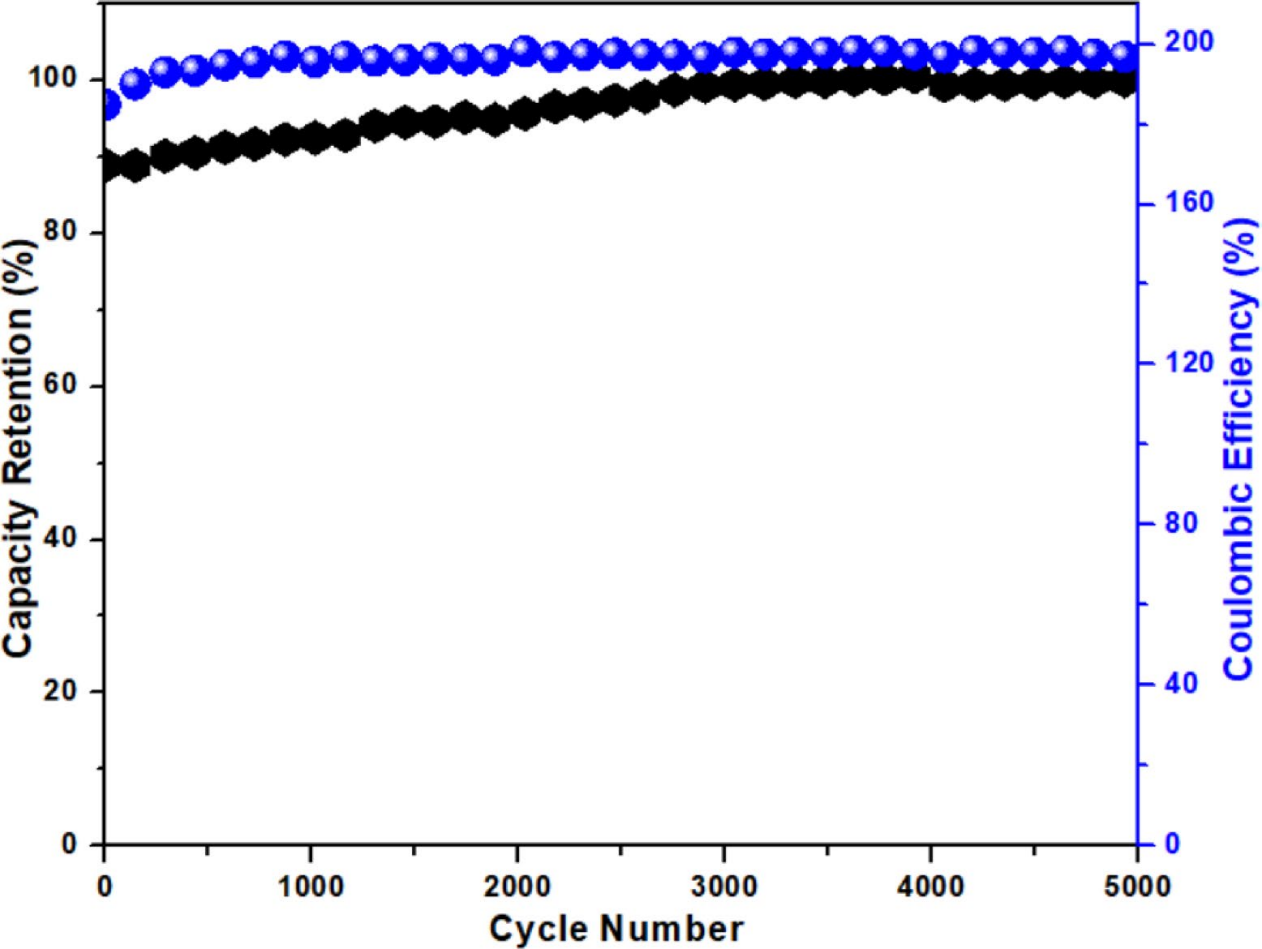
Cyclic stability of the Cu-WO_3_/MXene electrode measured over 5000 GCD cycles.

In this equation, ‘Cp’ represents the specific capacitance (Fg^− 1^), ‘Δt’ indicates the discharging time, and ‘ΔV’ is the potential window (V). To further evaluate the practical applicability of the electrode materials, a Ragone plot (energy density vs. power density) was constructed, as shown in Fig. 14. The Cu-WO_3_/MXene composite demonstrates a significantly enhanced energy density while maintaining high power density, indicating excellent electrochemical performance and suitability for high-power energy storage devices. Furthermore, the cyclic stability of the electrode was evaluated by performing GCD tests over 5000 cycles at a constant current density. As shown in Fig. 15, the Cu-WO_3_/MXene nanocomposite retained 89% of its initial capacitance after 5000 cycles, demonstrating excellent long-term electrochemical stability. The observed retention suggests structural integrity and strong interfacial bonding between Cu-WO_3_ and MXene, contributing to long-term performance. This stability can be attributed to the structural robustness of the composite and effective electron/ion transport facilitated by the MXene matrix.

**Table 4:** Specific capacitance (C_P_), energy density (E_D_), and power density (P_D_) values obtained from the galvanostatic charge-discharge curve.

Electroactive Material	Current Density (mA)	Specific Capacitance ‘C_*P*_’(F/g)	Energy Density ‘E_D_’(Wh/Kg)	Power Density ‘*P*_D_’ (W/Kg)
WO_3_	1	187.15	18.9	807.8
	2	174.22	17.6	1616.3
	3	157.4	15.93	2428
	4	135.37	13.70	3238.34
	5	123.22	12.47	4047.97
Cu-WO_3_ (5 at%)	1	243.27	24.63	809.95
	2	180.57	18.28	1619.6
	3	112.8	11.4	2425.53
	4	77.77	7.87	3238.88
	5	59.88	6.06	4047.77
Cu-WO_3_ (10 at%)	1	371.6	37.62	809.8
	2	362.4	36.69	1619.5
	3	254.8	25.79	2428.5
	4	201.15	20.36	3238.74
	5	179.55	18.17	4047.69
Cu-WO_3_ (15 at%)	1	564.6	57.16	809.8
	2	517.8	52.42	1619.7
	3	273.6	27.70	2429.8
	4	178.04	18.02	3238.90
	5	142.66	14.44	4047.79
CuWO_3_/MXene (15 at%)	1	692.4	70.10	809.8
	2	678.2	68.66	1619.7
	3	470	47.58	2426.17
	4	349.4	35.37	3238.92
	5	303.4	30.71	4047.84

**Table 5:** Comparison table with parameters calculated from electrochemical studies for various electrode materials.

Electrode Material	Specific Capacitance ‘C_*P*_’(F/g)	Energy Density ‘E_D_’(Wh/Kg)	Power Density ‘*P*_D_’ (W/Kg)	Specific capacitance retention	Reference
CoWO_4_/MXene	630 F g^− 1^ at 1 A g^− 1^ (1 M H_2_SO_4_ electrolyte)	63.8 Wh kg^− 1^	422 W kg^− 1^	92% over 10,000 cycles	^59^
WO_3_/MXene	376 F g^− 1^ at 0.5 A g^− 1^ (1 M H_2_SO_4_ aqueous electrolyte)	-	-	72% over 20,000 cycles	^60^
WO_3_/MXene	1930 F cm^− 3^ at 1 A g^− 1^	31 Wh kg^− 1^	650 W kg^− 1^	107% after 5000 cycles	^61^
CoWO_3_	792 F g ^− 1^ at 1 A g^− 1^(1.0 M AlCl_3_)	35.0 Wh kg-^1^	801	98.8%, 10,000 cycles	^62^
CuWO_3_/MXene	692.4 F g^− 1^ at 1 A g^− 1^ (1 M KOH electrolyte)	70.10 Wh kg^− 1^	809.8 W kg^− 1^	89% after 5,000 cycles	This work

#### Electrochemical impedance spectroscopy (EIS) analysis

**Fig. 16:**
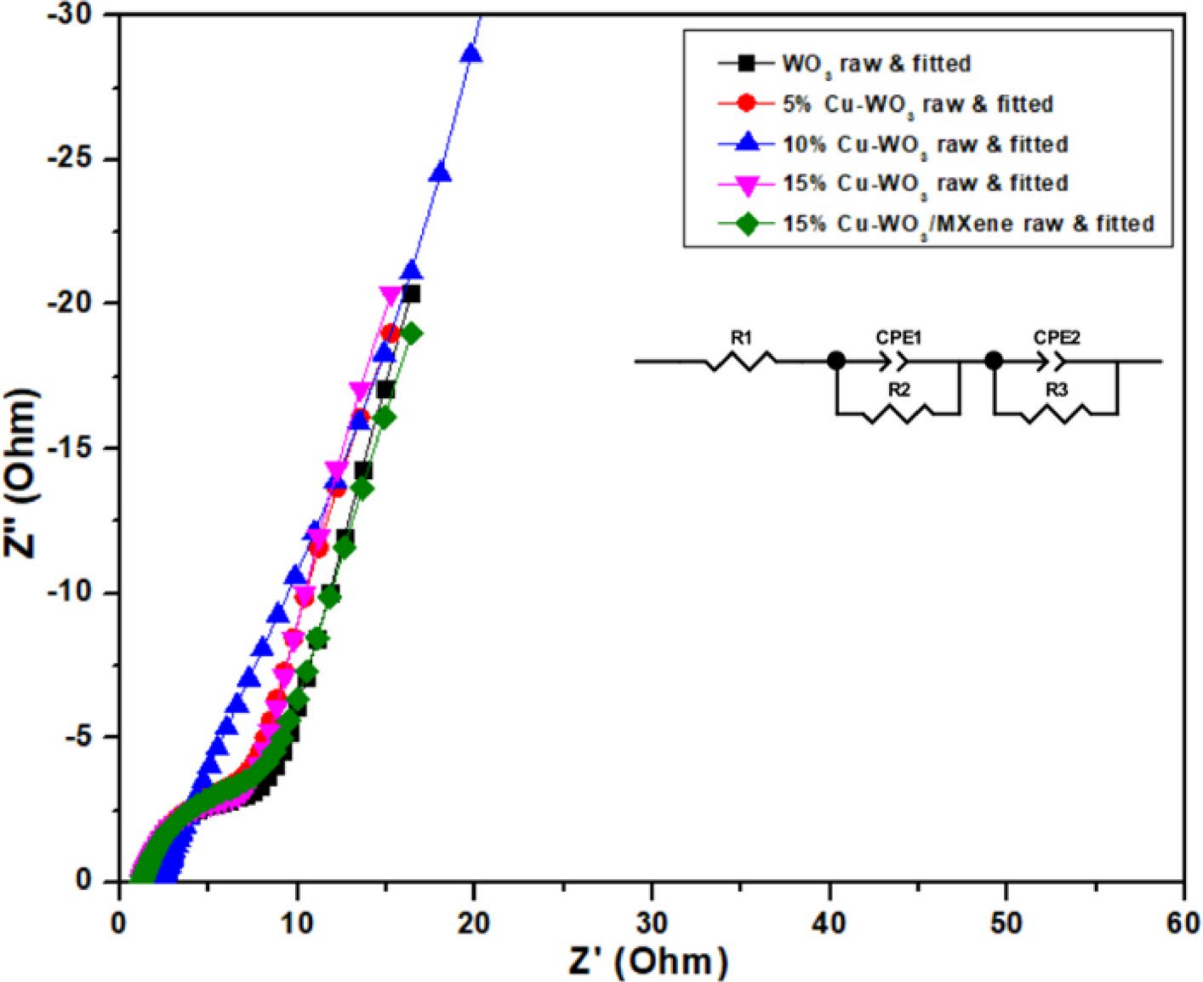
EIS plots of WO_3_, Cu-WO_3_ (Cu x% =5 at%, 10 at%, 15 at%), and 15 at% Cu-WO_3_/MXene nanocomposite, and the inset image shows the electrical equivalent circuit diagram.

Electrochemical impedance spectroscopy, or EIS analyses, was performed to assess the charge transmission capabilities of the WO_3_, Cu-WO_3_ (Cu x% = 5 at.%, 10 at.%, and 15 at.%), and 15% Cu-WO_3_/MXene electrodes. Figure 16 presents the outcomes of the prepared electrode assessments, which have been carried out at frequency intervals ranging from 100 kHz to 10 MHz^63^. The EIS measurements of the synthesized materials have been performed in a 1 M potassium hydroxide solution (KOH). The Nyquist plot depicts a semicircle at higher frequencies with a straight line that follows a semicircle at lower frequencies. Further, the three main sections of a Nyquist plot typically indicate the values of the three resistances that were measured during the EIS measurements. The real and imaginary impedance sections were denoted by Z’ and Z” in the Nyquist figure. The solution resistance (Rs) is represented by the high-frequency intercept on the real Z′ axis, accounting for the intrinsic resistance of the electrolyte and contact interface. The charge transfer resistance (Rct) is associated with the semicircular arc and reflects the resistance to faradaic charge transfer processes at the electrode surface. An additional resistance component (R3) is included in the model to represent diffusion-related or deeper interfacial resistance typically arising in porous electrode materials. The two CPE elements (CPE1 and CPE2) represent the non-ideal capacitive behavior of the double-layer and electrode surface, which deviates from ideal capacitors due to surface roughness and porosity. The EIS spectra were fitted using a modified Randles circuit containing Rs, Rct, two CPE elements, and an additional R3 to account for deeper interfacial or diffusion resistance is illustrated in the inset image of Fig. 16. The fitted parameters, including solution resistance (Rs), charge transfer resistance (Rct), and double-layer capacitance (Cdl) for Cu-WO_3_ and Cu-WO_3_/MXene electrodes, derived from Nyquist plot fitting, are summarized in Table 6. Cdl values were calculated from the CPE parameters using the Brug formula. Among the tested materials, the 15 at.% Cu-WO_3_/MXene nanocomposite exhibited the lowest charge transfer resistance Rct, indicating faster interfacial electron transfer and highest Cdl, confirming its superior charge transfer kinetics and interfacial capacitive behavior due to synergistic effects of Cu doping and MXene integration^64^.

**Table 6:** Fitted electrochemical impedance spectroscopy (EIS) parameters of Cu-WO_3_ and Cu-WO_3_/MXene electrodes, including solution resistance (Rs), charge transfer resistance (Rct), constant phase element parameters (CPE2-T and CPE2-n), and calculated double-layer capacitance (Cdl).

Sample	Rs (Ω)	Rct (Ω)	CPE2-T (F·sⁿ^− 1^)	CPE2-*n*	Cdl (F)
WO_3_	2.336	26.250	0.00352	0.9282	0.0029
Cu-WO_3_ (5 at%)	1.909	15.665	0.02632	0.9113	0.0165
Cu-WO_3_ (10 at%)	1.471	10.203	0.04722	0.8876	0.0393
Cu-WO_3_ (15 at%)	1.199	7.672	0.06652	0.8594	0.0582
CuWO_3_/MXene(15 at%)	1.176	6.619	0.06971	0.8018	0.0597

## Conclusion

In this study, we have proposed an easily performed co-precipitation approach for the fabrication of WO_3_ and Cu-WO_3_ (Cu x% = 5 at%, 10 at%, and 15 at%), as well as a facile sonication technique to create their composite with MXene (15at.% Cu-WO_3_/MXene). XRD, FESEM, and HRTEM investigations were used to ascertain the synthesized material’s structural and morphological characteristics. FTIR spectroscopy was employed to assess the functional groups. The elemental composition was investigated using XPS. The as-fabricated material’s charge-discharge storage characteristics were examined using CV, GCD, and EIS methodologies. Utilizing a three-electrode setup, the electrochemical experiments were performed in a 1 M KOH solution. When compared to the WO_3_ and Cu-WO_3_ (Cu x% = 5 at%, 10 at%, and 15 at%) electrodes, the fabricated 15 at% Cu-WO_3_/MXene electrode exhibits an exceptional specific capacitance of 692.4 F/g at 1 A/g, with excellent rate capability and long-term cycling stability, retaining 89% capacitance over 5000 cycles. This is owing to the synergistic impact of Cu-WO_3_ and MXene, which permits ions to easily diffuse into the material, enhancing electrochemical performance. Furthermore, Electrochemical impedance spectroscopy (EIS) analysis revealed that the 15 at% Cu-WO_3_/MXene electrode exhibited the lowest solution resistance (Rs = 1.176 Ω) and charge transfer resistance (Rct = 6.619 Ω), indicating enhanced conductivity and interfacial kinetics. These improvements are attributed to the synergistic effects of Cu doping and MXene integration, which together facilitate faster electron and ion transport. Consequently, the synthesized composite material is efficient for the forthcoming generation of energy-storing devices due to the remarkable supercapacitive performance demonstrated by the findings. Also, compared to previous reports on Cu-WO_3_ and MXene-based supercapacitors, the current composite system demonstrates superior performance, attributed to the synergistic effect of Cu doping and MXene incorporation. These findings confirm the potential of the material as a promising electrode candidate for next-generation energy storage devices.

## Data Availability

The raw/processed data required to reproduce these findings cannot be shared at this time as the data also forms part of an ongoing study and are available from the corresponding author on reasonable request.

## References

[1] Yi, S., Raza Abbasi, K., Hussain, K., Albaker, A. & Alvarado, R. Environmental concerns in the united states: can renewable energy, fossil fuel energy, and natural resources depletion help? *Gondwana Res.* **117**, 41–55 (2023).

[2] Khan, S. & Hossain, M. K. Classification and properties of nanoparticles. *Nanoparticle-Based Polym. Compos.* (Elsevier), 15–54. 10.1016/B978-0-12-824272-8.00009-9 (2022).

[3] Jie, H., Khan, I., Alharthi, M., Zafar, M. W. & Saeed, A. Sustainable energy policy, socio-economic development, and ecological footprint: The economic significance of natural resources, population growth, and industrial development. *Util. Policy*. **81**, 101490 (2023).

[4] Jadhav, A. L., Jadhav, S. L., Mandlekar, B. K. & Kadam, A. V. Hydrothermally synthesized three-dimensional hierarchical CuO nanomaterials for energy storage applications. *Mater. Chem. Phys.* **310**, 128494 (2023).

[5] Nur-E-Alam, M. et al. Multifunctional prospects of physical vapor-deposited silver-based metal-dielectric nanocomposite thin films. *J. Sci. Adv. Mater. Devices*. **10**, 100871 (2025).

[6] Darwish, M. A. et al. Enhancing electromagnetic interference mitigation: A comprehensive study on the synthesis and shielding capabilities of polypyrrole/cobalt ferrite nanocomposites. *Sustain. Mater. Technol.* **42**, e01150 (2024).

[7] Oyedotun, K. O. & Mamba, B. B. New trends in supercapacitors applications. *Inorg. Chem. Commun.* **170**, 113154 (2024).

[8] Hossain, M. K. et al. A review on recent applications and future prospects of rare Earth oxides in corrosion and thermal barrier coatings, catalysts, tribological, and environmental sectors. *Ceram. Int.* **48**, 32588–32612 (2022).

[9] Hossain, M. K. et al. A review on experimental and theoretical studies of perovskite barium zirconate proton conductors. *Emergent Mater.* **4**, 999–1027 (2021).

[10] Hossain, M. K. et al. A review of applications, prospects, and challenges of Proton-Conducting zirconates in electrochemical hydrogen devices. *Nanomaterials* **12**, 3581 (2022). 36296771 10.3390/nano12203581 PMC9609721

[11] Chakma, R. et al. Recent applications, challenges, and future prospects of microbial fuel cells: A review. *Glob Challenges* **9**, (2025). 10.1002/gch2.202500004 PMC12065106 40352631

[12] Hossain, M. K. et al. Prospects and challenges of proton conducting cerates in electrochemical hydrogen devices for clean energy systems: A review. *Glob Challenges*. 10.1002/gch2.202500119 (2025). 10.1002/gch2.202500119 PMC12246738 40656213

[13] Dissanayake, K. & Kularatna-Abeywardana, D. A review of supercapacitors: Materials, technology, challenges, and renewable energy applications. *J. Energy Storage*. **96**, 112563 (2024).

[14] Sriram, G. et al. Recent trends in hierarchical electrode materials in supercapacitor: Synthesis, electrochemical measurements, performance and their charge-storage mechanism. *J. Energy Storage*. **94**, 112454 (2024).

[15] Rashid Khan, H., Latif Ahmad, A. & Supercapacitors Overcoming current limitations and charting the course for next-generation energy storage. *J. Ind. Eng. Chem.* **141**, 46–66 (2025).

[16] Khandare, L. N., Gunjal, K., Bhosale, S. R., Bankar, P. & Chaure, N. B. High-performance supercapacitors based on Sb2Se3-rGO composite materials for advanced energy storage. *J. Alloys Compd.* **1021**, 179504 (2025).

[17] Khandare, L. N. et al. Facile synthesis and first principles calculations of Li-MoS2/rGO nanocomposite for high-performance supercapacitor applications. *J. Energy Storage*. **102**, 114166 (2024).

[18] Khandare, L. & Late, D. J. MoO3-rGO nanocomposites for electrochemical energy storage. *Appl. Surf. Sci.* **418**, 2–8 (2017).

[19] Khandare, L. N., Late, D. J. & Chaure, N. B. MoS2 nanobelts-carbon hybrid material for supercapacitor applications. *Front Chem* **11**, (2023). 10.3389/fchem.2023.1166544 PMC10477701 37674526

[20] Ammar, A. U. et al. All-in-one supercapacitor devices based on nanosized Mn4+-doped WO3. *J. Energy Storage*. **72**, 108599 (2023).

[21] Dhamodharan, D., Dhinakaran, V., Byun, H. S. & MXenes An emerging 2D material. *Carbon N Y*. **192**, 366–383 (2022).

[22] Kewate, O. J. & Punniyakoti, S. Ti3AlC2 MAX phase and Ti3C2TX MXene-based composites towards supercapacitor applications: A comprehensive review of synthesis, recent progress, and challenges. *J. Energy Storage*. **72**, 108501 (2023).

[23] Akhoondi, A. et al. Recent advances in the synthesis of ZnO-based electrochemical sensors. *Synth. Sinter.* **3**, 259–274 (2023).

[24] Kunwar, J. et al. Cobalt oxide decorated 2D mxene: A hybrid nanocomposite electrode for high-performance supercapacitor application. *J. Electroanal. Chem.* **950**, 117915 (2023).

[25] Rosaiah, P. et al. Carbon based manganese oxide (MnO2, MnO2/MWCNT and MnO2/rGO) composite electrodes for high-stability Li-ion batteries. *Carbon Lett.* **34**, 215–225 (2024).

[26] Yu, H., Ji, B. & Wang, D. Synthesis of Cu-WO3-CNT nanocomposite and its electrochemical and photocatalytic properties: Application for Cr(VI) removal from aqueous solution. *Int. J. Electrochem. Sci.* **18**, 100247 (2023).

[27] Jenila, T. J. et al. Exploring the synergistic photocatalytic performance and optical properties of NiMoO4 decorated Ti3C2Tx (NiMoO4/MXene) nanocomposite via hydrothermal technique. *Inorg. Chem. Commun.* **172**, 113679 (2025).

[28] Kotasthane, V. et al. Selective etching of Ti 3 AlC 2 MAX phases using quaternary ammonium fluorides directly yields Ti 3 C 2 T z MXene nanosheets: implications for energy storage. *ACS Appl. Nano Mater.* **6**, 1093–1105 (2023).

[29] Bejjanki, D. & Puttapati, S. K. PANI/WO3/MXene nanocomposite material for high-performance solid state symmetric supercapacitor. *Diam. Relat. Mater.* **146**, 111248 (2024).

[30] Rathod, V. T. et al. Hydrothermally synthesized nickel doped MnO2 nanocrystals for high-performance supercapacitor electrodes. *Inorg. Chem. Commun.* **166**, 112643 (2024).

[31] Rahman, M. T. et al. Fe2O3 nanoparticles dispersed unsaturated polyester resin based nanocomposites: effect of gamma radiation on mechanical properties. *Radiat. Eff. Defects Solids*. **174**, 480–493 (2019).

[32] Rahman, M. T. et al. Study on the mechanical, electrical and optical properties of metal-oxide nanoparticles dispersed unsaturated polyester resin nanocomposites. *Results Phys.* **13**, 102264 (2019).

[33] Rahman, M. T. et al. Evaluation of thermal, mechanical, electrical and optical properties of metal-oxide dispersed HDPE nanocomposites. *Mater. Res. Express*. **6**, 85092 (2019).

[34] Hoque, M. A. et al. Fabrication and comparative study of magnetic Fe and α-Fe2O3 nanoparticles dispersed hybrid polymer (PVA + Chitosan) novel nanocomposite film. *Results Phys.* **10**, 434–443 (2018).

[35] Ponelakkia, D. K. et al. Enhanced pseudocapacitive performance of transition metal doped MoO3 nanorods. *Electrochim. Acta*. **511**, 145349 (2025).

[36] Warsi, A. Z. et al. Synthesis and characterisation of WO 3 and ag 2 O nanoparticles and their nanocomposite for photocatalytic degradation of dyes. *Int. J. Environ. Anal. Chem.* **104**, 2755–2775 (2024).

[37] Zhang, K. et al. Uniaxial tensile test and theoretical analysis of a composite of high strength steel wire mesh and polyurethane cement. *Matéria (Rio Janeiro)* **29**, (2024).

[38] Kondalkar, V. V. et al. Nanobrick-like WO3 thin films: Hydrothermal synthesis and electrochromic application. *Superlattices Microstruct.* **73**, 290–295 (2014).

[39] Krishnaiah, P., Awan, H. T. A., Walvekar, R. & Manickam, S. MXene-Based composites and their applications. in 53–86 (2022). 10.1007/978-3-031-05006-0_4

[40] Haroon, A. & Ahmed, A. S. An insight into the microstructural properties and dielectric behavior of Ce doped WO3 nanoparticles. *Phys. B Condens. Matter*. **657**, 414798 (2023).

[41] Nishad, H. S. et al. Temperature-driven enhancement in pseudocapacitive charge storage of Sn-doped WO3 nanoflowers and its high-performance quasi-solid-state asymmetric supercapacitor. *J. Energy Storage*. **77**, 109842 (2024).

[42] Yang, L. et al. Methylene blue functionalized 3D MXene aerogel: Self-assembly by interfacial electrostatic interaction for asymmetric supercapacitors. *Electrochim. Acta*. **467**, 143088 (2023).

[43] Thakur, A. et al. Synthesis of a 2D tungsten MXene for electrocatalysis. *Nat. Synth.*10.1038/s44160-025-00773-z (2025).

[44] Park, H. W. & Roh, K. C. Recent advances in and perspectives on pseudocapacitive materials for Supercapacitors–A review. *J. Power Sources*. **557**, 232558 (2023).

[45] Dong, X., Lu, Y., Liu, X., Zhang, L. & Tong, Y. Nanostructured tungsten oxide as photochromic material for smart devices, energy conversion, and environmental remediation. *J. Photochem. Photobiol C Photochem. Rev.* **53**, 100555 (2022).

[46] Jindal, T., Phogat, P., Shreya, Singh, S. & Jha, R. Electrochemical and optical comparison of Cr3+, Co2+, Ag1+, Hg1 + and Pb4 + doped WO3 as a thin layer working electrode for electrochemical sensing. *Appl. Phys. A*. **130**, 512 (2024).

[47] Liu, L. et al. Dual active sites over TiO2 homojunction through tungsten doping and oxygen vacancies for enhanced photoelectrochemical properties. *J. Alloys Compd.* **962**, 171193 (2023).

[48] Nabeel, M. I., Hussain, D., Ahmad, N., Najam-ul-Haq, M. & Musharraf, S. G. Recent advancements in the fabrication and photocatalytic applications of graphitic carbon nitride-tungsten oxide nanocomposites. *Nanoscale Adv.* **5**, 5214–5255 (2023). 37767045 10.1039/d3na00159h PMC10521255

[49] Hajirnis, S. et al. Hydrothermal synthesis of WO 3 film on rough surface to analyze methanol gas at room temperature. *Mater. Res. Express*. **8**, 095503 (2021).

[50] Buddiga, L. R., Gajula, G. R. & T, S. R. Investigation of structural, chemical formation and oxidation States of Cu-doped Titania and WO3 co-doped Titania nanocomposite for antibacterial activity. *J. Sol-Gel Sci. Technol.*10.1007/s10971-024-06601-8 (2024).

[51] Luo, W. et al. Flexible Ti3C2Tx MXene/V2O5 composite films for high-performance all-solid supercapacitors. *J. Energy Storage*. **62**, 106807 (2023).

[52] Irfan, S. et al. Validating superior electrochemical properties of Ti 3 C 2 MXene for supercapacitor applications through first-principles calculations. *New. J. Chem.* **48**, 4982–4994 (2024).

[53] Pol, A. et al. Catalytic enhancement and energy dynamics of Cu-doped NiO on 304SS: mechanisms of oxygen evolution reaction at reduced activation energies. *Mol. Catal.* **576**, 114929 (2025).

[54] Nikhil, P., Vasanth, S., Ponpandian, N. & Viswanathan, C. Synthesis effect on surface functionalized Ti3C2Tx MXene supported nickel oxide nanocomposites with enhanced specific capacity for supercapacitor application. *J. Energy Storage*. **72**, 108414 (2023).

[55] Sohan, A., Banoth, P., Aleksandrova, M., Nirmala Grace, A. & Kollu, P. Review on < scp > mxene synthesis, properties, and recent research exploring electrode architecture for supercapacitor applications. *Int. J. Energy Res.* **45**, 19746–19771 (2021).

[56] Raj, R. et al. Enhanced electrochemical performance of nickel Oxide-Doped MXene heterostructures for advanced energy storage and conversion applications. *Fuel* **385**, 133916 (2025).

[57] Li, C. et al. Preparation of rGO/MXene@NiCo-P and rGO/MXene@Fe2O3 positive and negative composite electrodes for high-performance asymmetric supercapacitors. *J. Energy Storage*. **56**, 105986 (2022).

[58] Xi, W. et al. Hierarchical mxene/transition metal oxide heterostructures for rechargeable batteries, capacitors, and capacitive Deionization. *Nanoscale* **14**, 11923–11944 (2022). 35920652 10.1039/d2nr02802f

[59] Vigneshwaran, J. et al. Engineering the electrochemical performance of CoWO4 composites of MXene by transitional metal ion doping for high energy density supercapacitors. *J. Mater. Sci.* **59**, 10953–10970 (2024).

[60] Nishad, H. S. et al. Temperature driven pseudocapactive performance of WO3/MXene nanocomposite for asymmetric aqueous supercapacitors. *Chem. Eng. J.* **495**, 153360 (2024).

[61] Zhang, P. et al. Microporous tungsten oxide spheres coupled with Ti 3 C 2 T x nanosheets for high-volumetric capacitance supercapacitors. *Nanotechnology* **35**, 495401 (2024). 10.1088/1361-6528/ad6c55 39111321

[62] Thalji, M. R., Ali, G. A. M., Shim, J. J. & Chong, K. F. Cobalt-doped tungsten suboxides for supercapacitor applications. *Chem. Eng. J.* **473**, 145341 (2023).

[63] Madhavan, J. Transition metal oxides-MXene nanocomposite: the next frontier in supercapacitors. in 117–144 (2024). 10.21741/9781644903292-6

[64] Khan, A., Gaikwad, M. A., Kim, J. H. & Kadam, A. An overview and experimental analysis of WO3/TiO2 composite with enhanced electrochromic properties for smart windows application. *Tungsten* **6**, 732–747 (2024).

